# Gouqi-derived nanovesicles (GqDNVs) inhibited dexamethasone-induced muscle atrophy associating with AMPK/SIRT1/PGC1α signaling pathway

**DOI:** 10.1186/s12951-024-02563-9

**Published:** 2024-05-22

**Authors:** Xiaolei Zhou, Shiyin Xu, Zixuan Zhang, Mingmeng Tang, Zitong Meng, Zhao Peng, Yuxiao Liao, Xuefeng Yang, Andreas K. Nüssler, Liegang Liu, Wei Yang

**Affiliations:** 1 Department of Nutrition and Food Hygiene, Hubei Key Laboratory of Food Nutrition and Safety, Tongji Medical College, Huazhong University of Science and Technology, Hangkong Road 13, Wuhan, 430030 China; 2 Department of Nutrition and Food Hygiene and MOE Key Lab of Environment and Health, School of Public Health, Tongji Medical College, Huazhong University of Science and Technology, Hangkong Road 13, Wuhan, 430030 China; 3 Department of Traumatology, BG Trauma Center, University of Tübingen, Schnarrenbergstr. 95, 72076 Tübingen, Germany

**Keywords:** Aging, Goji, GqDNVs, Skeletal muscle, Metabolome analysis, AMPK

## Abstract

With the increasing trend of global aging, sarcopenia has become a significant public health issue. Goji berry, also known as “Gou qi zi” in China, is a traditional Chinese herb that can enhance the structure and function of muscles and bones. Otherwise, previous excellent publications illustrated that plant-derived exosome-like nanoparticles can exert good bioactive functions in different aging or disease models. Thus, we issued the hypothesis that Gouqi-derived nanovesicles (GqDNVs) may also have the ability to improve skeletal muscle health, though the effect and its mechanism need to be explored. Hence, we have extracted GqDNVs from fresh berries of *Lycium barbarum* L. (goji) and found that the contents of GqDNVs are rich in saccharides and lipids. Based on the pathway annotations and predictions in non-targeted metabolome analysis, GqDNVs are tightly associated with the pathways in metabolism. In muscle atrophy model mice, intramuscular injection of GqDNVs improves the cross-sectional area of the quadriceps muscle, grip strength and the AMPK/SIRT1/PGC1α pathway expression. After separately inhibiting AMPK or PGC1α in C2C12 cells with dexamethasone administration, we have found that the activated AMPK plays the chief role in improving cell proliferation induced by GqDNVs. Furthermore, the energy-targeted metabolome analysis in the quadriceps muscle demonstrates that the GqDNVs up-regulate the metabolism of amino sugar and nucleotide sugar, autophagy and oxidative phosphorylation process, which indicates the activation of muscle regeneration. Besides, the Spearman rank analysis shows close associations between the quality and function of skeletal muscle, metabolites and expression levels of AMPK and SIRT1. In this study, we provide a new founding that GqDNVs can improve the quality and function of skeletal muscle accompanying the activated AMPK/SIRT1/PGC1α signaling pathway. Therefore, GqDNVs have the effect of anti-aging skeletal muscle as a potential adjuvant or complementary method or idea in future therapy and research.

**Graphical Abstract:**

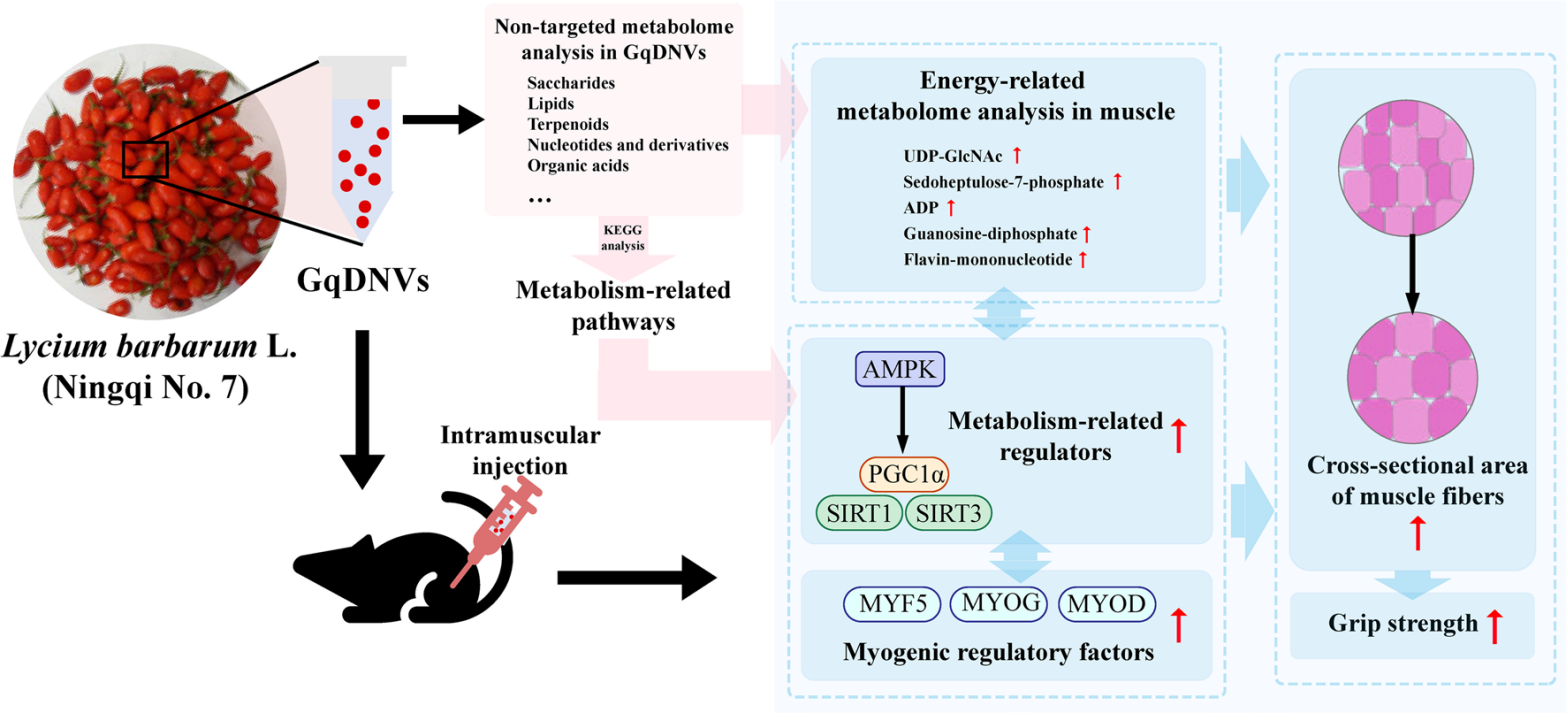

**Supplementary Information:**

The online version contains supplementary material available at 10.1186/s12951-024-02563-9.

## Background

With the development of the economy and society, people worldwide live longer, and the size and proportion of older people are also growing. By 2050, there will be 2.1 billion people aged 60 and over and 426 million aged 80 and over [1]. A distinctive feature of aging is the gradual loss of skeletal muscle mass and physical function associated with sarcopenia. Sarcopenia, a loss of skeletal muscle mass and function associated with aging, is a progressive and widespread skeletal muscle disease that can lead to disability, weakness and an increased medical burden [2]. Therefore, the world will face a severe social and economic burden with population aging. Sarcopenia will also become a health issue that we can not ignore, forcing us to urgently find effective methods to enhance skeletal muscle function and inhibit quality decline.

Goji, also known as “Gou qi zi” in China, is the ripening fruit of *Lycium barbarum* L. It was first published in “Sheng Nong’s Herbal Classic” and was listed as a top-grade herb with the function of delaying aging. There are 80 species of *Lycium* in the world and 7 species and 3 varieties in China, most of which are distributed in the northwest and north [3]. As a traditional Chinese herbal medicine for over 2000 years, goji is listed by the National Health Commission of China as a homologous species of medicine and food [4]. It is also a functional food in Asian countries due to its nutritional and medicinal value [5]. Goji is enriched in a variety of functional biochemicals and is effective in reducing cardiovascular risk [6], anti-inflammatory [7], improving energy metabolism [8], maintaining activities of antioxidants [9] and regulating immunity [10].

As a traditional Chinese herb, it is systematically recorded in the “Compendium of Materia Medica” written by Li Shizhen in 1578 that goji can strengthen the muscles and bones and has the effect of anti-fatigue and anti-aging. The extracts of goji can increase the mass of the tibialis anterior and the gastritis muscle and increase the average running distance of mice [11]. Goji extract and other bioactive components, such as betaine, can improve glucose uptake and adenosine triphosphate (ATP) production in C2C12 cells by up-regulating mitochondrial biogenic regulators [including peroxisome proliferator-activated receptor gamma coactivator1-alpha (PGC1α) and sirtuin 1 (SIRT1)] as well as activating AMP-activated protein kinase (AMPK) which can improve the energy metabolism and skeletal muscle function [12]. Current research has demonstrated that goji is able to strengthen skeletal muscle by altering energy metabolism in different conditions.

Exosomes are extracellular vesicles with a size range of 40 to 160 nm in diameter with an endosomal origin and contain nucleic acids, proteins, lipids, amino acids and other metabolites [13]. Plant-derived exosome-like nanoparticles are currently isolated from various squeezed fruits and vegetables and have been found with different components and functions similar to those of the original plants [14], which also have multiple biological effects, including anti-inflammatory [15, 16], promoting neural differentiation [17] and anti-tumor [18]. With the characteristics of a vast source, easy preparation, multi-component, low immune rejection and high bioavailability, plant-derived nanoparticles are also suitable as therapeutic means or drug delivery carriers in the medical field and have a strong application prospect [19]. Therefore, we hypothesized that gouqi-derived nanovesicles (GqDNVs) may have a fighting effect on muscle aging, while the impact and mechanism of GqDNVs on inhibiting muscle aging still need further research and exploration.

Regarding this point, we first used the ultracentrifuge and sucrose gradient method for receiving GqDNVs and utilized metabolome analysis to analyze contents and predict pathways. Meanwhile, we also used different platforms to figure out changes or responses in quadriceps muscle after GqDNVs injection in the dexamethasone-induced muscle atrophy mice model. Finally, we would provide a new method or idea for anti-skeletal muscle aging by improving muscle mass and function for future studies.

## Materials and methods

### GqDNVs preparation

**Fig. 1:**
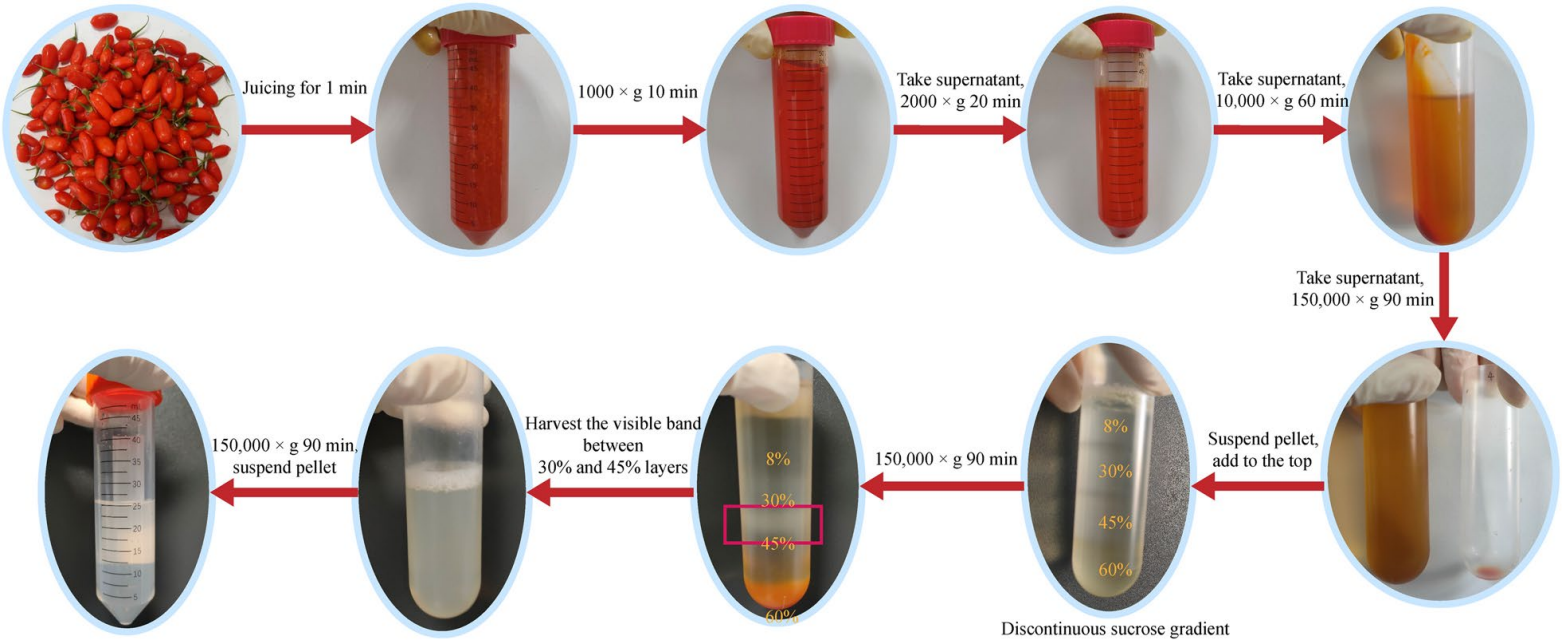
The extraction process of GqDNVs

GqDNVs preparation referred to publications with minor revisions [14, 20]. Fresh berries of *Lycium barbarum* L. (Ningqi No. 7) were collected from Yinchuan, Ningxia Province, China (106° 9′ 54.047″ E, 38° 39′ 11.498″ N, 1125 m above sea level). The berries were gently washed three times with deionized water and then put into the blender and juice for 1 min. Next, the fluid was centrifuged at 1000×*g* for 10 min, 2000×*g* for 20 min and 10,000×*g* for 60 min to remove fruit residues. The supernatant was then ultracentrifuged at 150,000×*g* for 1.5 h and the pellet was suspended in phosphate-buffered saline (PBS). For purification of GqDNVs, the suspension was transferred to a discontinuous sucrose gradient (8%/30%/45%/60%) and ultracentrifuged at 150,000×*g* for an additional 1.5 h [21–23]. The visible band between 30 and 45% layers was harvested, washed with PBS and then ultracentrifuged at 150,000×*g* for 1.5 h. The pellet was suspended with PBS and passed through with a 0.44 μm and a 0.22 μm filter, respectively. All procedures in this section are illustrated in Fig. 1. The suspension of GqDNVs was stored at − 80 °C for further experiments [24]. Then, GqDNVs were visualized using transmission electron microscopy (HT-7700, Hitachi, Japan) and characterizations (size and concentration) were determined by nanoparticle tracking analysis (NTA) using the Nanosight NS300 (Malvern Panalytical, England) [25].

### Cell culture and treatments

C2C12 cells were cultured in Dulbecco’s modified Eagle’s medium (DMEM; Gibco, Waltham, MA, USA) supplemented with 10% fetal bovine serum, 100 IU/mL penicillin and 0.1 mg/mL streptomycin in an incubator at 37 °C in a 5% of CO_2_ atmosphere. C2C12 cells were inoculated with a differentiation medium containing DMEM, 2% horse serum, 100 IU/mL penicillin and 0.1 mg/mL streptomycin for 5 days to differentiate into myotube. The primary culture methods and details are referred to in our published study [25].

### Cell viability

Cell counting kit-8 (CCK-8, Beyotime, China) was used to detect cell viability. The cells were seeded in 96-well plates at a 5 × 10^3^/well density in 100 μL medium for 24 h. The cells were then treated with different concentrations of GqDNVs (5 × 10^7^, 1 × 10^8^, 2.5 × 10^8^, 5 × 10^8^ and 1 × 10^9^ particles/mL) with or without 100 μM dexamethasone for 24 h. Then, CCK-8 reagent (10 μL) was added to each well and cultured for 2 h. Each intervention was repeated 6 times. The absorbance at 450 nm was measured by a microplate reader (Infinite M Nano, Tecan, Switzerland).

### Measurement of ATP in C2C12 cells

The C2C12 cells were seeded in 6-well plates in a 2 mL medium and divided into three groups (CON, DEX and DG). Each group has 5 wells. C2C12 cells were incubated with the intervention for 24 h in the CON group (PBS), DEX group (100 μM dexamethasone + PBS) and DG group (100 μM dexamethasone + 5 × 10^8^ particles/mL GqDNVs) after 5 days of differentiation. The concentration of dexamethasone used in C2C12 cells in this study referred to previous research [26]. The concentration of ATP in cells was measured by a commercial kit (Nanjing JianCheng Bioengineering Institute, China). The absorbance values were measured with a microplate reader (Infinite M Nano, Tecan, Switzerland).

### Measurement of mitochondrial membrane potential in C2C12 cells

The enhanced mitochondrial membrane potential assay kit with 5,5′,6,6′-Tetrachloro-1,1′,3,3′-tetraethyl-imidacarbocyanine iodide (JC-1, Beyotime, China) was utilized to estimate the impacts of dexamethasone and GqDNVs on mitochondrial membrane potential. The C2C12 cells were seeded in 6-well plates with 2 mL medium and divided into three groups (CON, DEX and DG). Each group has 3 wells. C2C12 cells were incubated with the intervention for 24 h in the CON group (PBS), DEX group (100 μM dexamethasone + PBS) and DG group (100 μM dexamethasone + 5 × 10^8^ particles/mL GqDNVs). After that, C2C12 cells were cocultured with JC-1 solution for 20 min at 37 °C. The fluorescences were detected with the inverted fluorescence microscope (Axio Observer 7, Zeiss, Germany). We measured the area of JC-1 monomers and aggregates in 4 fields per well (12 fields in each group) through Image J.

### Cellular uptake assay

GqDNVs were labeled with PKH 26 (red fluorescent, MedChemExpress, China). PKH 26 was dissolved in dimethylsulfoxide to obtain 1 mM stock solution of PKH 26. The stock solution was diluted with PBS at 1:100 to obtain the 10 μM working solution. GqDNVs were incubated with the working solution at room temperature for 30 min and then ultracentrifuged at 150,000×*g* for 1.5 h to obtain PKH 26 labeled-GqDNVs. The PKH 26 labeled-GqDNVs were incubated with C2C12 cells at 37 °C in darkness for 6 h. After the incubation, the cells were harvested and washed using PBS to remove free particles. Cytoskeletons of C2C12 cells were displayed with Fluorescein isothiocyanate (FITC) Phalloidin (green fluorescent, MaoKang, China) and the nucleus was labeled with 4′,6-diamidino-2-phenylindole (DAPI) (blue fluorescent, Beyotime, China). These dyes enable efficient and accurate tracking processes of cellular endocytosis of GqDNVs. The endocytosis of GqDNVs by C2C12 cells was observed by point scanning laser confocal (FV3000, OMTOOLS, China).

Fluorescence-activated cell sorting was also used to measure the quantity of C2C12 cells uptake PKH 26 labeled-GqDNVs. The C2C12 cells were seeded in 6-well plates with 2 mL medium and divided into three groups (Blank, Positive and GqDNVs). The C2C12 cells were incubated with the intervention for 6 h in the Blank group (PBS), Positive group (10 μM PKH 26) and GqDNVs group (PKH 26 labeled-GqDNVs). The quantity of C2C12 cells with positive fluorescence was measured by full spectrum analytical flow cytometry (ID 7000, SONY, Japan).

### Measurement of diameter of C2C12 cells

We used Jenner-Giemsa staining (Beyotime, China) to stain the tubes of C2C12 cells after differentiation. The C2C12 cells were observed with an inverted microscope. The diameter of the muscle tubes was measured with Image J software. In this section, C2C12 cells were inoculated in 6-well plates and divided into three groups (CON group, DEX group and DG group), and each group was inoculated with 5 wells. C2C12 cells were incubated with the intervention for 24 h in the CON group (PBS), DEX group (100 μM dexamethasone + PBS) and DG group (100 μM dexamethasone + 5 × 10^8^ particles/mL GqDNVs) after 5 days of differentiation. We measured the diameter of about 50 tubes per well (250 tubes in each group), and each myotube was measured 3 times to obtain its average value [25].

### The inhibition of AMPK and PGC1α pathways

Dorsomorphin dihydrochloride (Compound C dihydrochloride, 10 μM, MedChemExpress, China) [27] was used to inhibit the activity of AMPK and SR-18292 (20 μM, MedChemExpress, China) [28] was used to inhibit the activity of PGC1α, respectively. After 5 days of differentiation, C2C12 cells were incubated with the intervention for 18 h in the CON group (PBS), DEX group (100 μM dexamethasone), DG group (100 μM dexamethasone + 5 × 10^8^ particles/mL GqDNVs), CC group (10 μM Compound C dihydrochloride + 100 μM dexamethasone + 5 × 10^8^ particles/mL GqDNVs) and SR group (20 μM SR-18292 + 100 μM dexamethasone + 5 × 10^8^ particles/mL GqDNVs).

### Animals

The 7-week-old male C57BL/6J mice were purchased from Vital River (Beijing, China). Mice were housed under specific pathogen-free conditions. Animal care was performed according to the Institute for Laboratory Animal Research regulations, and all animal procedures were approved by the Institutional Animal Care and Use Committee, Huazhong University of Science and Technology (IACUC Number: 3626).

### In vivo image for GqDNVs distribution in mice after GqDNVs injection

GqDNVs were labeled by DiR (AAT Bioquest, USA). DiR was dissolved in dimethylsulfoxide to obtain a 1 mM stock solution of DiR. The stock solution was diluted with PBS at 1:200 to get the 5 μM working solution. GqDNVs were incubated with the working solution at room temperature for 20 min and then ultracentrifuged at 150,000×*g* for 1.5 h to obtain DiR-GqDNVs. C57BL/6J mice were injected with GqDNVs into the left quadriceps muscle. The mice in the CON group were injected with PBS. After 2, 8 and 24 h, GqDNVs distribution in alive mice, dissected quadriceps muscle, gastrocnemius muscle, tibialis anterior muscle and triceps muscle was determined with the live optical imaging systems of small animals (Lago X optical imaging systems; SI Imaging, USA). The fluorescent intensities in the region of interest (ROI) were measured by the Aura imaging software (SI Imaging, USA). The fluorescent intensity in vivo was compared with the CON group by the two-sample t-test. The fluorescent intensity in dissected muscle at different time points was analyzed by two-way analysis of variance (two-way ANOVA). The pairwise comparisons in multiple groups were analyzed using the Tukey multiple comparison test.

### Muscle atrophy model mice and treatment

After 7 days of adaptive feeding, C57BL/6J mice were randomly divided into 3 groups: CON group (normal mice + 50 μL PBS), DEX group (muscle atrophy mice + 50 μL PBS) and DG group (muscle atrophy mice + 50 μL GqDNVs), with 10 mice in each group. The C57BL/6 mice were administered intraperitoneal injection of 25 mg/kg d dexamethasone for 9 days to model muscle atrophy mice. Then, the mice were injected intraperitoneally 5 mg/kg d dexamethasone to maintain the state of muscle atrophy. The muscle atrophy model was based on previous research [29]. Then, GqDNVs (total number of GqDNV particles: 1 × 10^8^) or PBS (equal volume) was injected into the quadriceps muscle daily for 14 days.

### Grip strength test

A grip strength meter was used to measure the grip strength of mice. After setting the gauge to 0 g, we soothed the mouse and placed it horizontally on a grip plate. The mouse’s tail was then slowly pulled back until the mouse released the grip plate. Three consecutive tests were performed for each mouse, and the mean value was calculated. The grip strength of mice was normalized by body weight.

### Treadmill test and footprint analysis

A treadmill measured the running distance. The mice were acclimated to treadmill running before the test was performed. Mice ran uphill at 10° on the treadmill at a 10 m/min starting speed. After 2 min, the speed was increased by 2 m/min to a final speed of 20 m/min. The standard of exhaustion was determined as the inability of the animal to remain on the treadmill despite electrical prodding. The total running distance of the mice was recorded, which was regarded as the ability of the mice to move. Otherwise, the footprint analysis (stride length) is shown in the supplementary data (Supplementary methods).

### The cross-sectional area of muscle fibers in mice

The middle part of the quadriceps muscle was cut from mice in each group for H&E staining following the standard procedures. Through a microscope (100×), 100 muscle fibers were randomly selected from muscle cut in the cross-section of each mouse. The cross-sectional area of muscle fibers was calculated using Image J.

### Biochemical indicators of serum or muscle in mice

Serum samples were collected after centrifuging the clotted blood at 14,000×*g* for 15 min. The amount of lactate dehydrogenase (LDH) and creatine kinase (CK) in serum was determined using commercial kits (Nanjing JianCheng Bioengineering Institute, China). The contents of LDH and CK were measured with the microplate reader (Infinite M Nano, Tecan, Switzerland). The detecting methods of LDH, CK, antioxidant (superoxide dismutase, SOD) and lipid peroxidation indicators (malondialdehyde, MDA) in muscle have been shown in supplementary data (Supplementary methods).

### Western blotting

Muscle samples and cultured cells were lysed in radioimmunoprecipitation assay buffer (150 mM of NaCl; 0.1% SDS; 50 mM of Tris (pH 7.4); 1% Triton X-100; 1% sodium deoxycholate) (Beyotime, China) and lysates were cleared by centrifugation at 12,000×*g* for 15 min at 4 °C. The protein concentration was determined by using a BCA assay (Beyotime, China). Aliquots of proteins (loading quantity of protein sample: 40 μg) were separated on 10% SDS–polyacrylamide gels and transferred to polyvinylidene difluoride membranes. The membranes were blocked for 2 h at room temperature with 5% non-fat milk in Tris-buffered saline plus Tween 20 (TBST), followed by overnight incubation with the primary antibodies at 4 °C, including anti-Myogenin (1:1000, Abcam), anti-Myf5 (1:1000, Abcam), anti-Myod1 (1:1000, ABclonal), anti-PGC1α (1:1000, Abcam), anti-SirT3 (1:1000, Cell Signaling), anti-SirT1 (1:1000, ABclonal), anti-phosph-mTOR (1:1000, Cell Signaling), anti-mTOR (1:1000, Cell Signaling), anti-phosph-AMPKα (Thr172) (1:1000, Cell Signaling), anti-AMPKα (1:1000, Cell Signaling) and anti-β-actin (1:1000, Cell Signaling). After washing in TBST, the membranes were incubated for 1 h at room temperature with the appropriate secondary antibody conjugated to horseradish peroxidase. Each membrane was detected with an enhanced chemiluminescence reagent, and the signals were subsequently caught. Values were normalized to β-actin and calculated using Image J.

### Quantitative real-time polymerase chain reaction (Q-PCR)

Total RNA extraction was extracted from the quadriceps muscle and tibialis anterior muscle of mice with RNA Easy Fast Tissue/Cell Kit (TIANGEN, China), and cDNA was synthesized with the FastKing gDNA Dispelling RT SuperMix (TIANGEN, China). The Q-PCR was performed with the FastReal qPCR PreMix (SYBR Green) (TIANGEN, China) using Quant Studio™ 7 Flex (Applied Biosystems, USA). Reaction conditions: 95 °C for 2 min; 40 cycles of 95 °C for 5 s and 60 °C for 15 s. Each sample has three duplications, and the results were processed by ^ΔΔ^Ct method.

### Metabolome analysis

Contents of GqDNVs and metabolites of quadriceps muscle samples were detected by the MetWare platform. Non-targeted and saccharides-targeted metabolome analysis of GqDNVs was based on the UPLC–ESI–MS/MS system and the Agilent 8890-5977B platform. Targeted metabolome analysis of muscle samples was based on the AB Sciex QTRAP 6500 LC–MS/MS platform. Identified metabolites were annotated using the Kyoto Encyclopedia of Genes and Genomes (KEGG) compound database, and annotated metabolites were then mapped to the KEGG pathway database. The principal component analysis (PCA) and the orthogonal partial least squares-discriminant analysis (OPLS-DA) were used to show the variance between groups in the targeted metabolome analysis in muscle [30]. Variable importance in projection (VIP) value and fold change (FC) were used to find the differential metabolites between groups in the targeted metabolome analysis of muscle. VIP is based on the OPLS-DA model. FC is the ratio of the mean value in the experimental group to the mean value in the control group. The metabolites that meet these two conditions are considered as the differential metabolites: VIP > 1; FC ≥ 2 or FC ≤ 0.5. The KEGG pathways associated with the differential metabolites are used for the enrichment analysis. The *P*-value is calculated by the hypergeometric test. Differential abundance score (DA score) is calculated as follows: (the number of up-regulated differential metabolites in this pathway − the number of down-regulated differential metabolites in this pathway)/the number of all metabolites annotated to this pathway [31]. More details are provided in the supplementary data (Supplementary methods).

### Statistic analysis

GraphPad Prism for Windows (Version 9.0.0, GraphPad Software, San Diego, California USA, www.graphpad.com) was used for data analysis and charting. In this study, multiple groups (≥ 3 groups) were compared and analyzed by One-way analysis of variance (one-way ANOVA) and the pairwise comparisons in multiple groups were analyzed using the Tukey multiple comparison test. The bilateral t-test was used to compare the values between the two groups. *P*-values less than 0.05 were considered statistically significant.

## Results

### GqDNVs’ morphology was a “cup-disk” profile, and their contents were rich in saccharides and related to pathways in metabolism

**Fig. 2:**
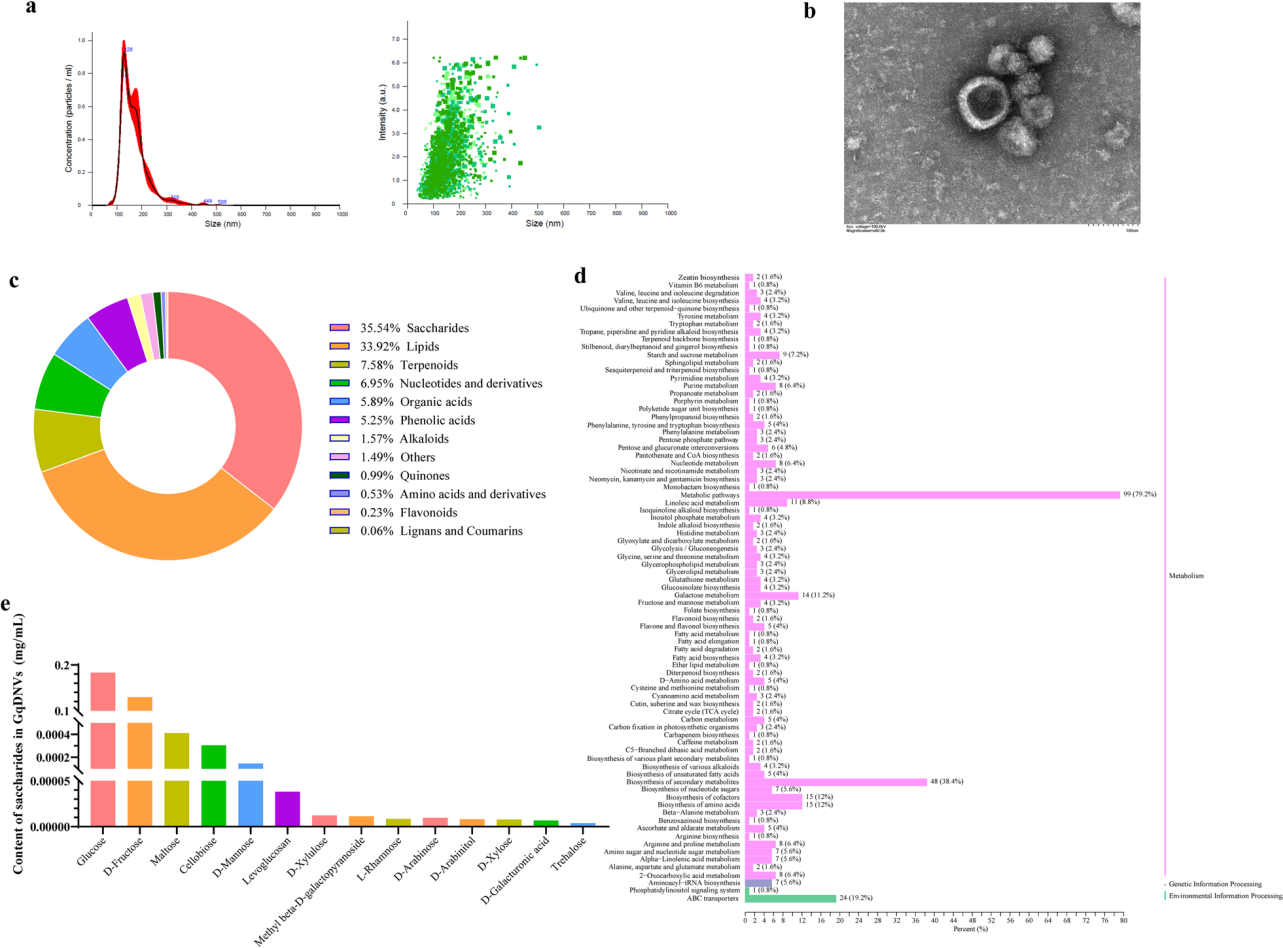
Identification of GqDNVs and metabolome analysis on GqDNVs. **a** The average concentration and particle size of GqDNVs were obtained by NTA. **b** Picture of GqDNVs under transmission electron microscopy. **c** Results of non-targeted metabolome analysis about GqDNVs. **d** KEGG classification of results based on non-targeted metabolome analysis in GqDNVs. **e** Results of saccharides-targeted metabolome analysis about GqDNVs

GqDNVs were extracted from fresh *Lycium barbarum* L. (Ningqi 7) by combining ultracentrifugation and density gradient centrifugation (Fig. 1). Through NTA and transmission electron microscopy, we found that GqDNVs were extracellular vesicles with a “cup-disk” shape at a size of 127.8 ± 1.3 nm (Fig. 2a, b). During NTA analysis, we found that GqDNVs showed irregular Brownian motion under the camera view (Supplementary Radio).

To explore the compositions and contents of GqDNVs, we first determined metabolites of GqDNVs by non-targeted metabolome analysis. We found that GqDNVs were rich in saccharides (35.54%) and lipids (33.92%) (Fig. 2c). Based on the KEGG pathway database, we found the contents of GqDNVs were associated with metabolism-related pathways, including biosynthesis of amino acids (ko01230), biosynthesis of cofactors (ko01240), metabolic pathways (ko01100) and biosynthesis of secondary metabolites (ko01110), and ATP-binding cassette transporter (ABC transporter) (ko02010) (Fig. 2d). Further, saccharides-targeted metabolome analysis revealed that GqDNVs contained different types and contents of monosaccharides and disaccharides, including glucose, d-fructose, maltose, cellobiose, d-mannose and other substances (Fig. 2e and Table 1).

**Table 1:** The concentration of saccharides in GqDNVs

Compounds	Class	Mean (mg/mL)	SD (mg/mL)
Glucose	Monosaccharide	1.83 × 10^–01^	5.81 × 10^–02^
d-Fructose	Monosaccharide	1.30 × 10^–01^	2.80 × 10^–02^
Maltose	Disaccharide	4.12 × 10^–04^	6.20 × 10^–05^
Cellobiose	Disaccharide	3.06 × 10^–04^	2.38 × 10^–04^
d-Mannose	Monosaccharide	1.45 × 10^–04^	5.28 × 10^–05^
Levoglucosan	Monosaccharide	3.81 × 10^–05^	1.41 × 10^–05^
d-Xylulose	Monosaccharide	1.22 × 10^–05^	6.15 × 10^–06^
Methyl beta-d-galactopyranoside	Monosaccharide	1.13 × 10^–05^	3.92 × 10^–06^
d-Arabinose	Monosaccharide	9.61 × 10^–06^	3.01 × 10^–06^
l-Rhamnose	Monosaccharide	8.41 × 10^–06^	4.87 × 10^–06^
d-Arabinitol	Monosaccharide	8.22 × 10^–06^	5.43 × 10^–06^
d-Xylose	Monosaccharide	7.90 × 10^–06^	1.34 × 10^–05^
d-Galacturonic acid	Monosaccharide	6.76 × 10^–06^	7.42 × 10^–06^
Trehalose	Disaccharide	3.93 × 10^–06^	9.63 × 10^–06^

The concentration of GqDNVs in the saccharides-targeted metabolome analysis was found to be 1 × 10^11^ particles/mL by nanoparticle tracking analysis

### GqDNVs could be absorbed by C2C12 cells and promote the diameter of myotubes in dexamethasone-administrated C2C12 cells

**Fig. 3:**
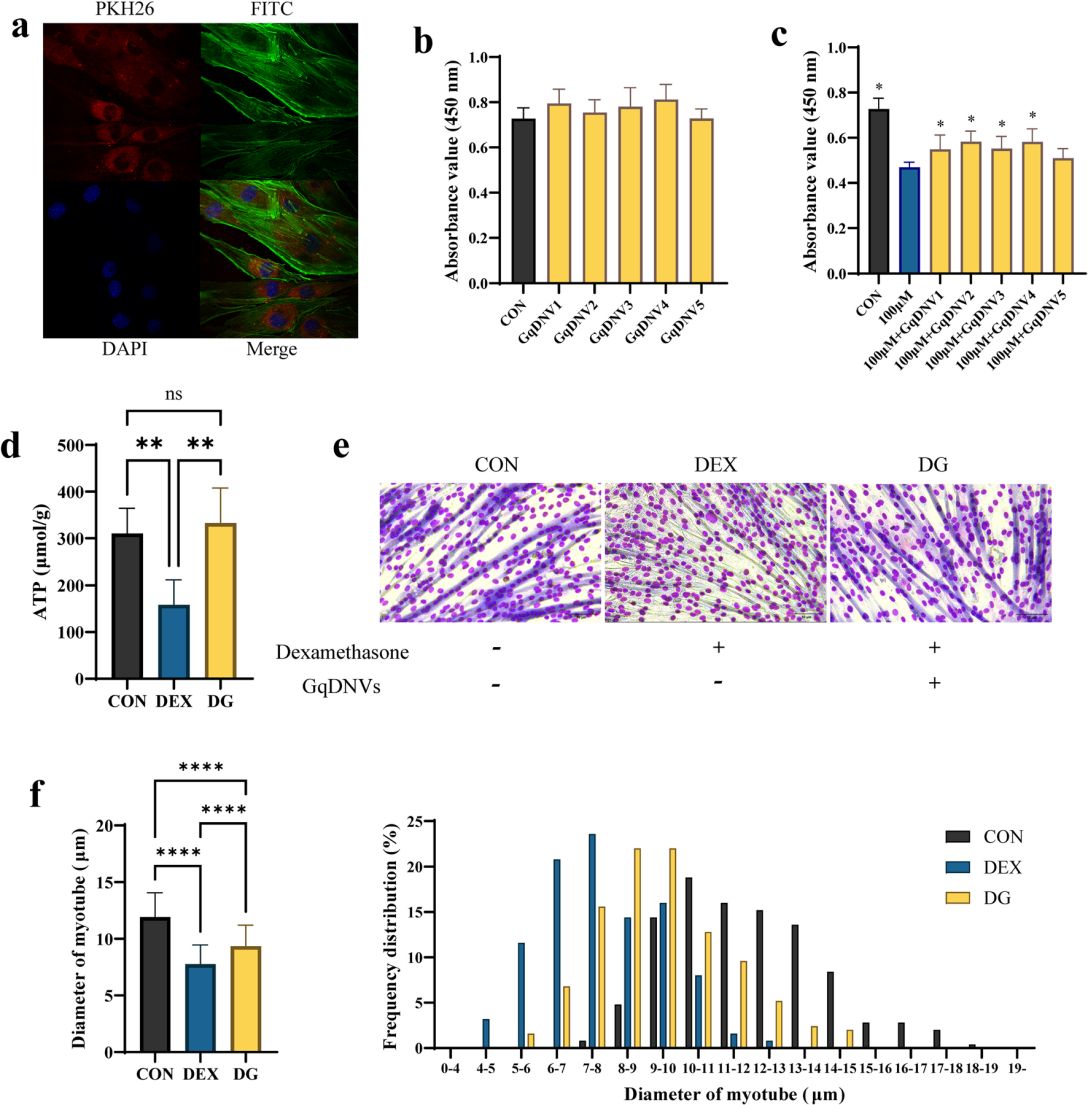
The effect of GqDNVs on C2C12 cells. **a** Picture of C2C12 cells and GqDNVs through fluorescence confocal microscopy. PKH 26 marked GqDNVs (red fluorescent); FITC stained cytoskeleton of C2C12 cells (green fluorescent); DAPI stained the nucleus of C2C12 cells (blue fluorescent); the merged diagram was a coincidence diagram of the three fluorescent images. **b**, **c** Cell viability test of GqDNVs. “CON” means C2C12 cells were treated with PBS; “100 μM” means the administration of 100 μM dexamethasone; “GqDNV1-5” means the administration of different concentrations of GqDNVs (1 × 10^9^, 5 × 10^8^, 2.5 × 10^8^, 1 × 10^8^, 5 × 10^7^ particles/mL). “*” indicates that there is a significant difference compared with the 100 μM dexamethasone intervention group (*P* < 0.05) after the two-sample t-test analysis. **d** The concentration of ATP in C2C12 cells. **e** The representative images of diameters of myotubes in C2C12 cells. Scale bars show 50 μm. **f** Quantitative analyses of the mean diameter of myotubes [mean ± standard deviation (SD)] and diameter distribution of myotubes of C2C12 cells. **d**–**f** One-way ANOVA analysis and Tukey’s multiple comparison analysis were used in the between-group comparisons. “*”, “**”, “***” and “****” indicate that after Tukey’s multiple comparison analysis, the* P*-value is lower than 0.05, 0.01, 0.001 and 0.0001

After co-culture of PKH 26-stained GqDNVs with C2C12 cells for 6 h, we first observed that GqDNVs were taken up by C2C12 cells through fluorescence confocal microscopy (Fig. 3a). The quantity of fluorescence-positive C2C12 cells was 56% by the fluorescence-activated cell sorting (Supplementary Fig S1). Besides, the results from the CCK-8 test showed that GqDNVs had no toxic effect on the cell viabilities of C2C12 cells (Fig. 3b), while GqDNVs could alleviate the toxicity of 100 μM dexamethasone on C2C12 cells (Fig. 3c). “GqDNV1-5” (Fig. 3b, c) means the administration of different concentrations of GqDNVs (successively 1 × 10^9^, 5 × 10^8^, 2.5 × 10^8^, 1 × 10^8^ and 5 × 10^7^ particles/mL). Based on the results from the CCK-8 test, cell viabilities in C2C12 cells with dexamethasone and GqDNVs (1 × 10^8^, 2.5 × 10^8^, 5 × 10^8^ and 1 × 10^9^ particles/mL) were significantly higher than in C2C12 cells with dexamethasone and the average absorbance value in C2C12 cells with dexamethasone and 5 × 10^8^ particles/mL GqDNVs was the highest in the groups (Fig. 3c). Therefore, we choose the 5 × 10^8^ particles/mL GqDNVs as the dosage for subsequent cell experiments.

After differentiation, C2C12 cells were treated with GqDNVs (5 × 10^8^ particles/mL) and 100 μM dexamethasone. The concentration of ATP in C2C12 cells in the DEX group was lower than in the CON group, while the GqDNVs increased the ATP in the DG group (Fig. 3d). To determine the effect of dexamethasone and GqDNVs on mitochondria, the mitochondrial membrane potential of the C2C12 cells was detected by JC-1 (Supplementary Fig S2). The results showed that dexamethasone reduced the mitochondrial membrane potential of C2C12 cells, while GqDNVs alleviated or retarded this decreasing trend. Moreover, the average myotube diameter of C2C12 cells in the DG group was significantly higher than in the DEX group (Fig. 3e, f). These results indicated that GqDNVs inhibited the atrophy and improved the metabolism of C2C12 cells under the dexamethasone condition.

### After injection into the quadriceps muscle, GqDNVs could be absorbed by the muscle

**Fig. 4:**
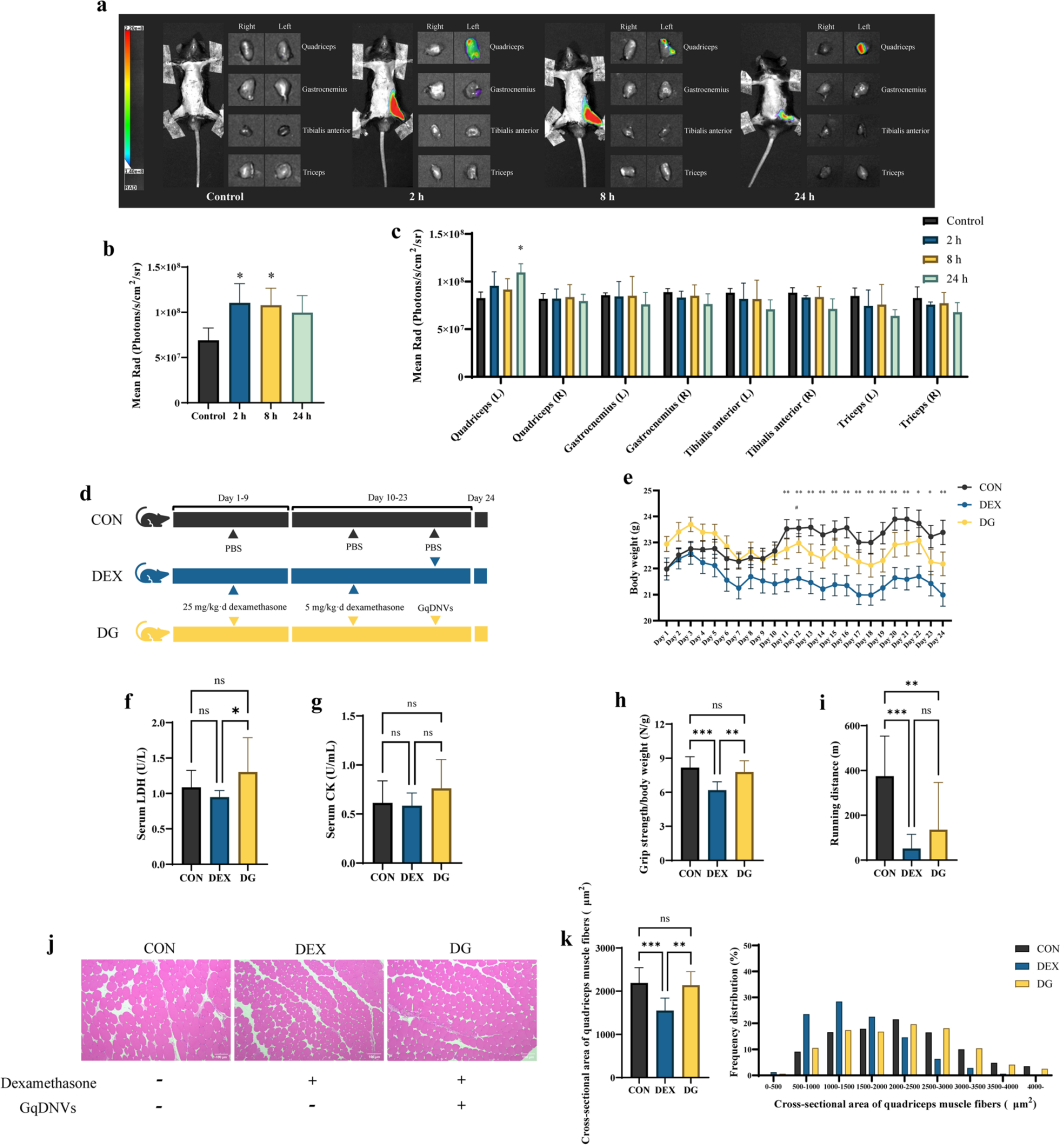
The effect of GqDNVs on C57BL/6J mice. **a** GqDNVs distribution in vivo. The mice were injected with GqDNVs labeled by DiR in the left quadriceps muscle. Images were taken 2 h, 8 h and 24 h after injections. The radiation dose was measured in photons. **b**, **c** The mean radiation dose in the left leg of mice and muscles (N = 3). **d** Grouping and intervention of C57BL/6J mice. N = 10 in each group. **e** The changes in body weight of mice in the 3 groups during the 24-day intervention (mean ± SE). Two-way ANOVA analysis (time × group) was used in the between-group comparisons and the factors of time, group and time × group were significant (*P* < 0.05). “*” and “**” indicate that after Tukey’s multiple comparison analysis, the* P*-value is lower than 0.05 and 0.01 in the comparison between the CON group and DEX group; “#” indicates the* P*-value is lower than 0.05 in the comparison between the DG group and DEX group. **f**, **g** Concentration of LDH and CK in serum (mean ± SD). **h** The ratio of grip strength to body weight in 3 groups (mean ± SD). **i** The running distance of mice in 3 groups (mean ± SD). **j** The representative images of the cross-sectional area of quadriceps muscle fibers. Scale bars show 100 μm. **k** Quantitative analyses of mean cross-sectional area (mean ± SD) and cross-sectional area distribution of quadriceps muscle fibers. **f**–**k** One-way ANOVA analysis and Tukey’s multiple comparison analysis were used in the between-group comparisons. “*”, “**”, “***” and “****” indicate that after Tukey’s multiple comparison analysis, the* P*-value is lower than 0.05, 0.01, 0.001 and 0.0001

Moreover, we detected the GqDNVs distribution in mice. After being injected with DiR-labeled GqDNVs in the left quadriceps muscle, the images were taken after 2 h, 8 h and 24 h injection and then the muscles were dissected (Fig. 4a–c). The fluorescent intensities were higher in the 2 h and 8 h groups compared with the control group (Fig. 4b). The results from two-way ANOVA analysis also showed a difference in fluorescent intensities of dissected muscles between the control group and 2 h, 8 h and 24 h groups. The fluorescent intensity of the left quadriceps muscle in the 24 h group was higher than the control group, while there were no differences in other muscles (Fig. 4c). It was indicated that GqDNVs were absorbed by the quadriceps muscle, while the GqDNVs were confined to the injected quadriceps muscle and rarely escaped.

### GqDNVs could alleviate dexamethasone-induced skeletal muscle atrophy and improve skeletal muscle function in mice

The information on group and intervention methods is shown in Fig. 4d. After 9 days with dexamethasone intervention (25 mg/kg d), the weight (Supplementary Fig S3) was not different in groups. However, the grip strength (Supplementary Fig S4) and running distance (Supplementary Fig S5) of mice in the DEX and DG groups were significantly lower than those in the CON group after a 9-day dexamethasone intervention. It was indicated that the muscle atrophy model was successful.

The body weight of mice was not different in between-group comparisons on days 1–10, while the body weight in the DEX group was lower than the CON group on days 11–24 (Fig. 4e). After the intervention of GqDNVs (on day 24), we also measured the activity of LDH (Fig. 4f) and CK (Fig. 4g) in serum. No differences were found between the CON and DEX groups, while the LDH in the DG group was higher than in the DEX group. Besides, the activities of LDH, CK and SOD and the content of MDA in muscle were not different between groups (Supplementary Fig S6–9).

The weight of quadriceps muscle (Supplementary Fig S10) and average stride length (Supplementary Fig S11) were not different among the three groups. Moreover, with the administration of dexamethasone, the grip strength (Fig. 4h), running distance (Fig. 4i), and cross-sectional area of quadriceps muscle fibers (Fig. 4j, k) in the DEX group were lower than in the CON group. However, the intervention of GqDNVs alleviated the reduction in grip strength and the cross-sectional area of quadriceps muscle fibers induced by dexamethasone in the DG group. Compared with the DEX group, the running distance of mice in the DG group demonstrated an upward pattern but was not noticeable. These results showed that GqDNVs can inhibit muscle strength loss in dexamethasone-induced muscle atrophy mice.

### GqDNVs affected the expression levels of myogenic factor 5 (MYF5), myogenin (MYOG) and myogenic differentiation (MYOD) proteins in muscle, which was associated with AMPK/SIRT1/PGC1α pathways

**Fig. 5:**
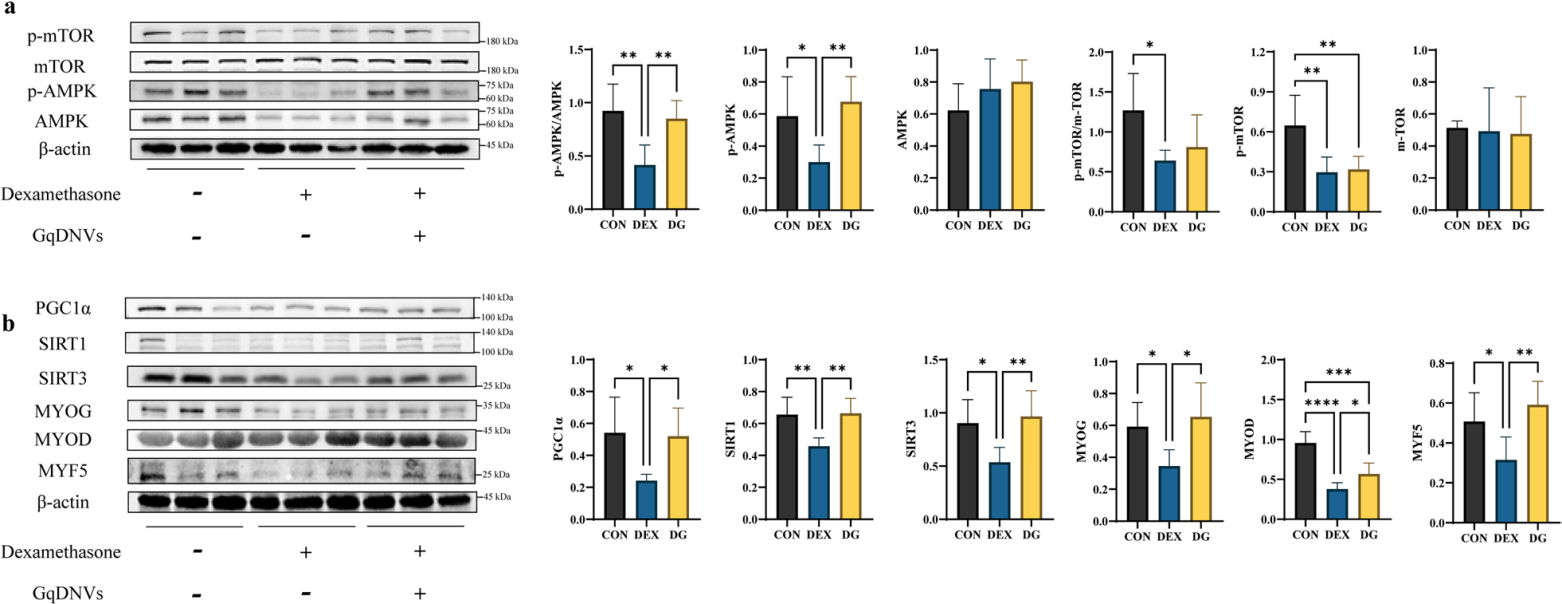
The results of the effect of GqDNVs on quadriceps muscle of mice through Western blotting. **a** The left panels show representative Western blotting of p-mTOR, mTOR, p-AMPK, AMPK and β-actin in the quadriceps muscle of mice. The right panels show quantitative analyses of p-AMPK/AMPK, p-AMPK, AMPK, p-mTOR/mTOR, p-mTOR and mTOR signals (mean ± SD). All the signals were normalized to the β-actin signal. N = 6 in each group. **b** The left panels show representative Western blotting of PGC1α, SIRT1, SIRT3, MYF5, MYOG, MYOD and β-actin in the quadriceps muscle of mice. The right panels show quantitative analyses of PGC1α, SIRT1, SIRT3, MYF5, MYOG and MYOD signals (mean ± SD). All the signals were normalized to the β-actin signal. N = 6 in each group. **a**, **b** One-way ANOVA analysis and Tukey’s multiple comparison analysis were used in the between-group comparisons. “*”, “**”, “***” and “****” indicate that after Tukey’s multiple comparison analysis, the* P*-value is lower than 0.05, 0.01, 0.001 and 0.0001

Furtherly, we investigated the molecular mechanism of the muscle improvement effect of GqDNVs on skeletal muscles. We found that the contents in GqDNVs were rich in saccharides and lipids, which were predicted and related to metabolism through the non-targeted metabolome analysis of GqDNVs. GqDNVs alleviated the reduction in grip strength and the cross-sectional area of quadriceps muscle fibers induced by dexamethasone in this study. Therefore, we assessed the protein expressions of the critical regulators involved in muscle health and metabolism: SIRT1, sirtuin 3 (SIRT3), PGC1α, mTOR and AMPK. Dexamethasone induced decreases in the protein-expressing levels of p-AMPK/AMPK, SIRT1, SIRT3 and PGC1α in the quadriceps muscle tissues of mice, while GqDNVs increased the protein levels in the DG group compared with the DEX group. However, the expression level of p-mTOR/mTOR was decreased by dexamethasone but not influenced by GqDNVs. The expressions of myogenic regulatory factors MYF5, MYOD and MYOG were also inhibited in the muscle atrophy mice, but GqDNVs could alleviate this pattern (Fig. 5).

Q-PCR analysis also demonstrated that the dexamethasone exposure induced down-regulation of AMPK, SIRT1, PGC1α and muscle differentiation factors, including MYF5, MYOD and MYOG, while GqDNVs alleviated the changes (Supplementary Fig S12). Moreover, Q-PCR for myosin heavy chain isoforms showed dexamethasone increased the myosin heavy chain 7 (MYH7) mRNA expression level (Supplementary Fig S13) in quadriceps muscle and decreased the mRNA expression levels of myosin heavy chain 2 (MYH2) and myosin heavy chain 4 (MYH4) in tibialis anterior muscle (Supplementary Fig S14), which indicated that dexamethasone induced the fast-to-slow myosin heavy chain isoform transition in muscle. GqDNVs recovered the dexamethasone-induced increase of the MYH7 mRNA expression level in the quadriceps muscle (Supplementary Fig S13) and insignificantly increased the MYH4 mRNA expression level in the tibialis anterior muscle (Supplementary Fig S14), which indicated that GqDNVs slightly alleviated the fast-to-slow myosin heavy chain isoform transition in muscle.

### The AMPK pathway might play an essential role in the muscle health effect of GqDNVs

**Fig. 6:**
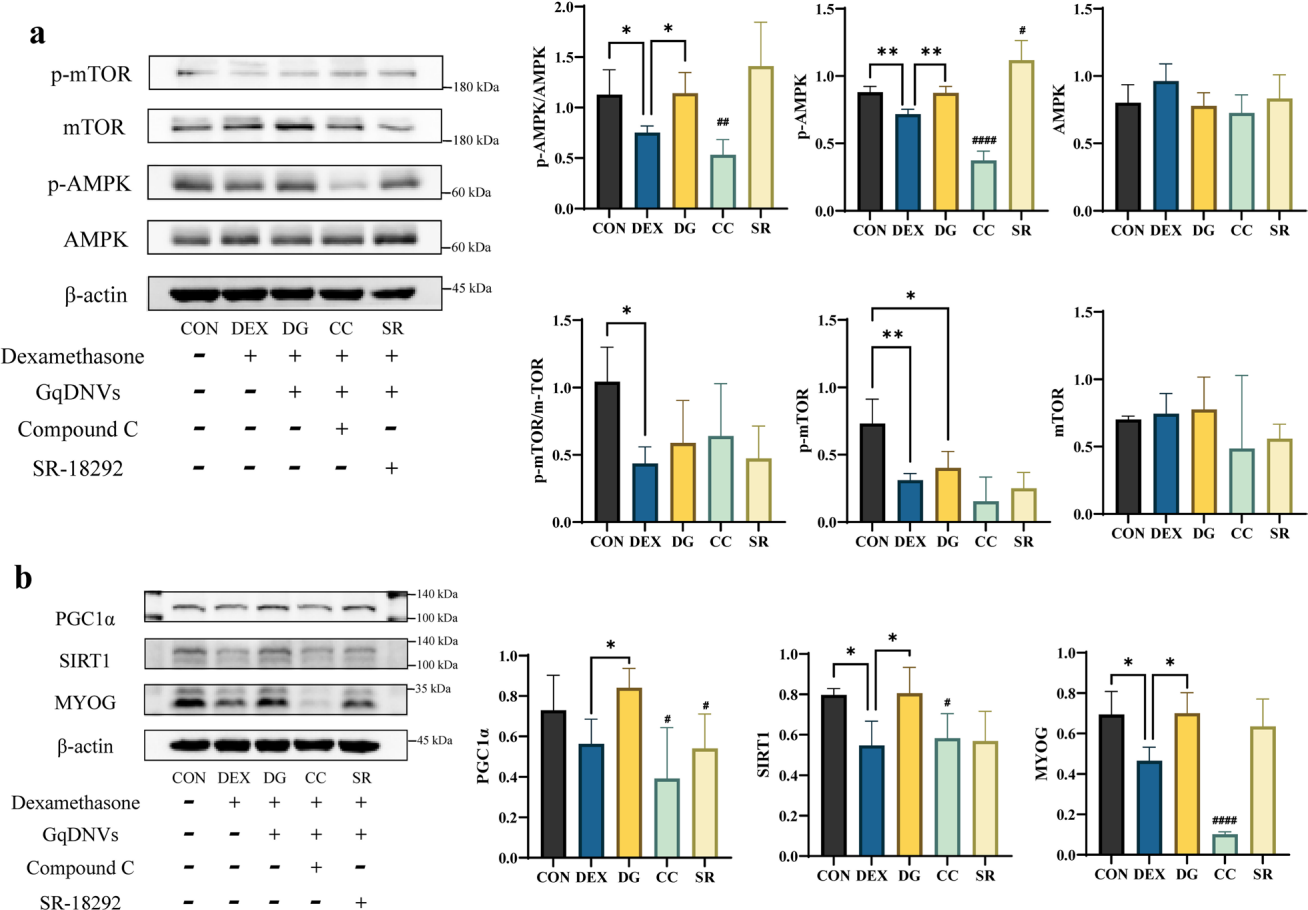
The results of the effect of GqDNVs on C2C12 cells through Western blotting. **a** The top left panels show representative Western blotting of p-mTOR, mTOR, p-AMPK, AMPK and β-actin in C2C12 cells. The right panels show quantitative analyses of p-AMPK/AMPK, p-AMPK, AMPK, p-mTOR/mTOR, p-mTOR and mTOR signals (mean ± SD). All the signals were normalized to the β-actin signal. N = 4 in each group. **b** The top left panels show representative images of PGC1α, SIRT1, MYOG and β-actin in Western blotting. The right panels show quantitative analyses of PGC1α, SIRT1 and MYOG signals (mean ± SD). All the signals were normalized to the β-actin signal. N = 4 in each group. **a**, **b** One-way ANOVA analysis and Tukey’s multiple comparison analysis were used to compare CON, DEX and DG groups. CC group and SR group were compared with the DG group by the t-test analysis. “*”, “**”, “***” and “****” indicates that after Tukey’s multiple comparison analysis, the* P*-value is lower than 0.05, 0.01, 0.001 and 0.0001. “#”, “##”, “###” and “####” indicate that after the t-test analysis, the* P*-value is lower than 0.05, 0.01, 0.001 and 0.0001

The changes in p-AMPK/AMPK, PGC1α, SIRT1 and MYOG induced by dexamethasone and GqDNVs in C2C12 cells had a similar pattern to the changes in the quadriceps muscle. To testify whether the AMPK or PGC1α mediated the effect of GqDNVs on skeletal muscle, we inhibited the activity of AMPK and PGC1α in C2C12 cells, respectively, using compound C dihydrochloride (AMPK inhibitor) and SR-18292 (PGC1α inhibitor). Compared with the DG group, the expressions of p-AMPK/AMPK, PGC1α, SIRT1 and MYOG in the CC group were significantly reduced by compound C dihydrochloride, but the p-mTOR/mTOR was unaffected. After the inhibition of PGC1α, the expression of PGC1α in the SR group was lower than in the DG group, while the other protein levels were not different between the SR and DG groups. The expression of p-AMPK in the SR group was higher than in the DG group, while there were no differences between the DG and SR groups in p-AMPK/AMPK, p-mTOR/mTOR, SIRT1 and MYOG (Fig. 6).

### GqDNVs changed the metabolites in the quadriceps muscle and the alterations were associated with the activated metabolic pathways

According to the results of non-targeted metabolome analysis in GqDNVs, the main components of GqDNVs were saccharides and lipids which were associated with the metabolism based on the KEGG pathway database. Therefore, to explore the molecular mechanism in the effect of GqDNVs on muscle further, we detected energy-related metabolites in the quadriceps muscle of mice in the DEX group and DG group with the targeted metabolome analysis.

**Fig. 7:**
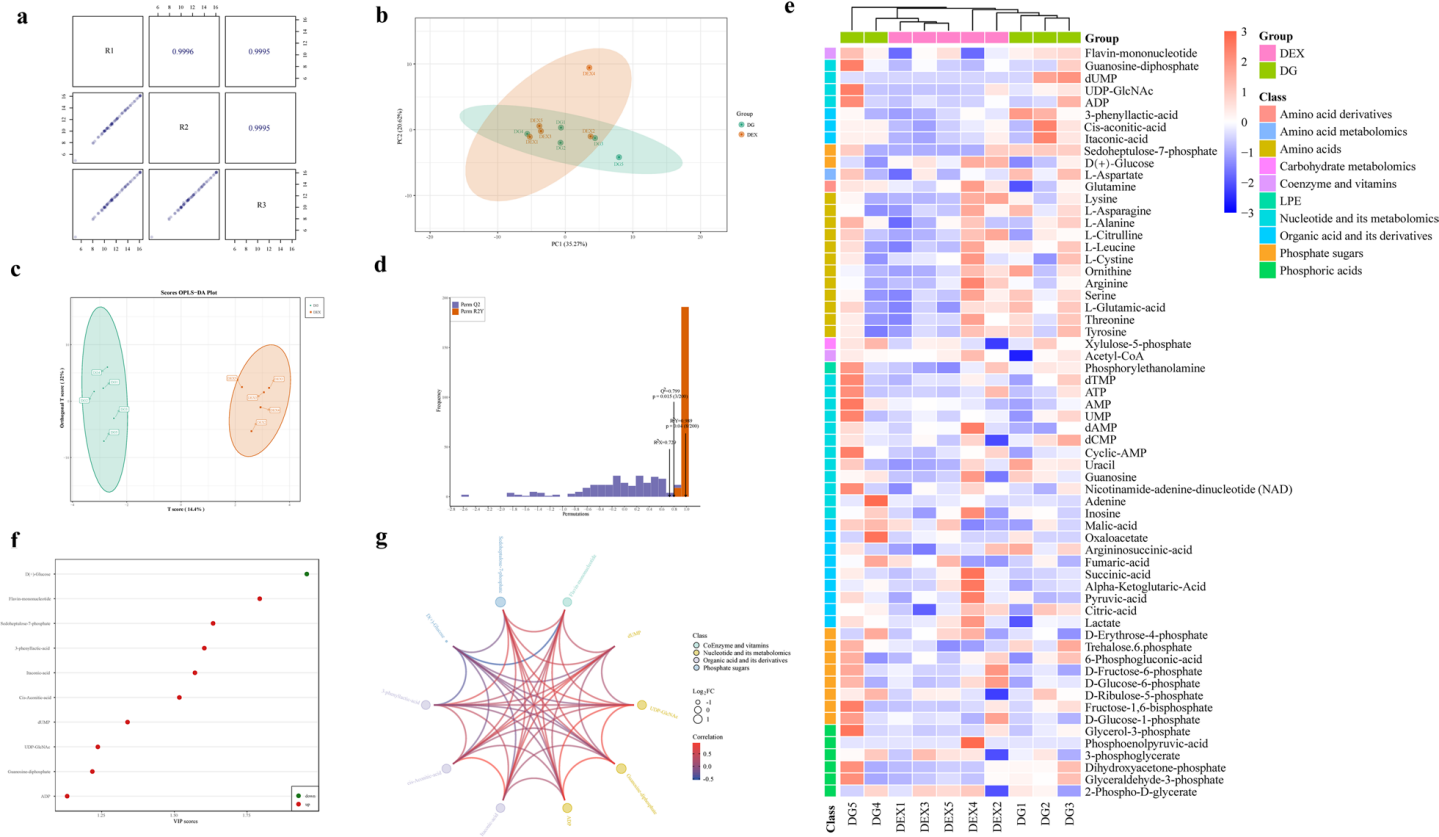
The energy-targeted metabolome analysis on quadriceps of mice. **a** Quality control (QC) samples correlation analysis. The square in the lower-left corner of the diagonal is the scatter diagram of the correlation of QC samples; the horizontal and vertical coordinates are metabolite content; each point in the figure represents a metabolite. The square in the upper right corner of the diagonal is the correlation coefficient of the corresponding QC samples. **b** PCA of the differences in metabolites between the DEX and DG groups. PC1 and PC2 represent the first and the second principal components, respectively. The percentage represents the interpretation rate of that principal component to the data set. **c** OPLS-DA model of energy-related metabolites in DEX group and DG group. The horizontal coordinate represents the principal component of prediction and shows the difference between groups. The ordinate represents the orthogonal principal component and shows the difference within the group. Percentages represent the degree of component that explains the data set. **d** The validation of the OPLS-DA model. The horizontal coordinate represents the R^2^Y and Q^2^ values in the model. The vertical coordinate is the frequency of the model classification effect in 200 random permutation and combination experiments. Orange represents the R^2^Y (randomization model), purple represents the Q^2^ (randomization model), and the black arrows represent the values of the original model (R^2^X, R^2^Y and Q^2^). **e** The heatmap of the energy-related metabolites in the quadriceps muscle in DEX and DG groups. The content of differential metabolites was conducted by unit variance scaling. The original data is centralized and divided by the standard deviation of the variable. **f** VIP value map of differential metabolites between DEX group and DG group. **g** The circos diagram of differential metabolite. “FC” means the fold change of the differential metabolite

**Fig. 8:**
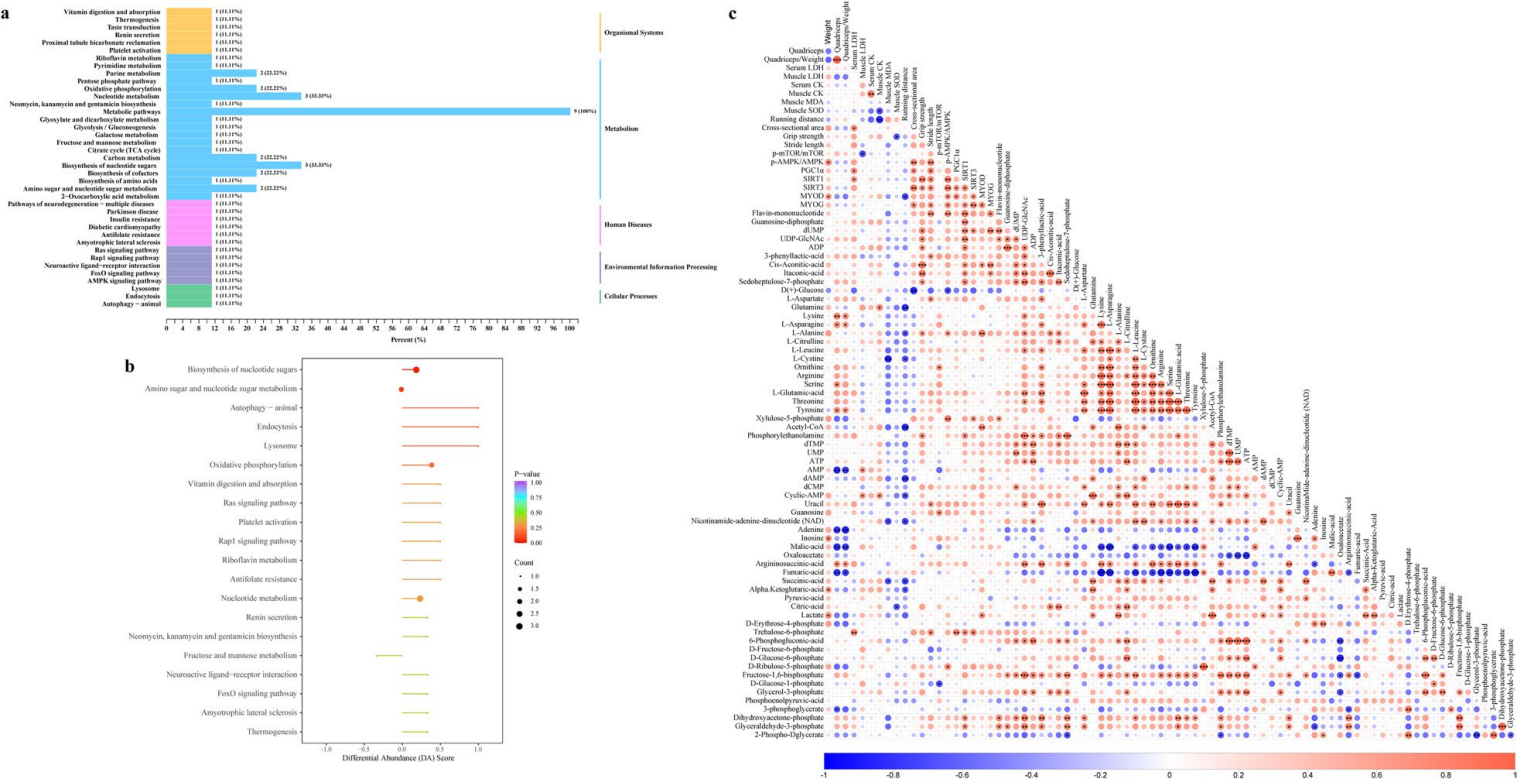
The associations between the metabolites and quality and function of muscle in mice. **a** KEGG classification of differential metabolites between DEX group and DG group. **b** The enrichment analysis of KEGG pathways related to differential metabolites. DA score is calculated as follows: (the number of up-regulated differential metabolites in this pathway—the number of down-regulated differential metabolites in this pathway) / the number of all metabolites annotated to this pathway. The size of the dots in the figure represents the number of differential metabolites enriched to the pathway. The *P*-value is calculated by the hypergeometric test. **c** The heatmap of correlations in differential metabolites and other indicators in mice through the Spearman rank test. “*”, “**” and “***” indicate that after the Spearman rank test, the* P*-value is lower than 0.05, 0.01 and 0.001

The correlation analysis of quality control samples demonstrated that the energy-related metabolome analysis was reliable (Fig. 7a). Through the representation of PCA (Fig. 7b), OPLS-DA (Fig. 7c, d) and heatmap (Fig. 7e), there were differences in energy-related metabolites between the DEX and DG groups. The concentrations of metabolites are shown in Table 2. We found that flavin-mononucleotide, guanosine-diphosphate, deoxyuridine monophosphate (dUMP), Uridine diphosphate N-acetylglucosamine orchestrates (UDP-GlcNAc), adenosine diphosphate (ADP), 3-phenyllactic-acid, cis-aconitic-acid, itaconic-acid and sedoheptulose-7-phosphate in DG group were higher compared with the DEX group, based on VIP value and FC. However, glucose in the DG group was lower than in the DEX group (Fig. 7f and Table 2). The differential metabolites had close associations with each other (Fig. 7g). Meanwhile, the related pathways of the differential metabolites were also classified according to the KEGG annotation results (Fig. 8a). Based on the DA score, we found that the metabolism-related pathways, especially biosynthesis of nucleotide sugars (ko01250), amino sugar and nucleotide sugar metabolism (ko00520), autophagy (ko04140), endocytosis (ko04144) and lysosome (ko04142), were up-regulated in the DG group compared with the DEX group (Fig. 8b). It was indicated that the GqDNVs activated the metabolism in skeletal muscle. The KEGG analysis of contents in GqDNVs also demonstrated that the metabolites in GqDNVs were closely associated with pathways in metabolism, including biosynthesis of nucleotide sugars (ko01250) and amino sugar and nucleotide sugar metabolism (ko00520) which were also shown in the targeted metabolome analysis of the quadriceps muscle of mice.

**Table 2:** The energy-related metabolites in the quadriceps muscle of mice in DEX and DG groups

Compounds	Class	DEX group (ng/g)	DG group (ng/g)	Fold change	VIP value	Type
l-Aspartate	Amino acid metabolomics	71,682.81 ± 12,513.30	87,658.96 ± 14,938.83	1.22	1.40	insig
Glutamine	Amino acid derivatives	24,434.66 ± 3795.65	21,778.21 ± 4229.35	0.89	0.94	insig
Lysine	Amino acids	30,095.70 ± 14,959.24	26,373.75 ± 6620.81	0.88	0.45	insig
l-Asparagine	Amino acids	5929.43 ± 2383.18	6323.99 ± 2152.73	1.07	0.25	insig
l-Alanine	Amino acids	46,527.00 ± 7287.81	52,340.62 ± 5500.85	1.12	1.23	insig
l-citrulline	Amino acids	2370.42 ± 512.63	2302.79 ± 339.08	0.97	0.15	insig
l-Leucine	Amino acids	34,962.79 ± 12,359.44	39,175.12 ± 8468.07	1.12	0.57	insig
l-Cystine	Amino acids	13.98 ± 9.64	10.03 ± 10.19	0.72	0.65	insig
Ornithine	Amino acids	649.32 ± 370.01	734.78 ± 385.49	1.13	0.34	insig
Arginine	Amino acids	22,945.74 ± 7361.00	19,449.89 ± 2654.26	0.85	0.86	insig
Serine	Amino acids	17,066.45 ± 6597.93	18,131.22 ± 4825.18	1.06	0.27	insig
l-Glutamic-acid	Amino acids	69,130.24 ± 20,978.77	87,003.74 ± 20,171.8	1.26	1.14	insig
Threonine	Amino acids	36,912.10 ± 10,915.28	38,793.56 ± 8407.82	1.05	0.27	insig
Tyrosine	Amino acids	59,925.79 ± 18,831.67	63,295.12 ± 21,934.34	1.06	0.22	insig
Xylulose-5-phosphate	Carbohydrate metabolomics	60,846.94 ± 24,322.97	84,485.71 ± 15,679.36	1.39	1.43	insig
Flavin-mononucleotide	Coenzyme and vitamins	68.63 ± 64.90	141.42 ± 24.63	2.06	1.79	up
Acetyl-CoA	Coenzyme and vitamins	132.93 ± 24.80	104.08 ± 58.67	0.78	0.91	insig
Phosphorylethanolamine	Lysophosphatidyl ethanolamine	15,014.38 ± 4333.78	18,977.86 ± 4207.78	1.26	1.22	insig
dTMP	Nucleotide and its metabolomics	155.62 ± 17.71	183.41 ± 60.06	1.18	0.86	insig
Guanosine-diphosphate	Nucleotide and its metabolomics	224.25 ± 240.90	771.23 ± 759.55	3.44	1.22	up
ATP	Nucleotide and its metabolomics	6220.79 ± 6069.73	8626.78 ± 9028.83	1.39	0.46	insig
UMP	Nucleotide and its metabolomics	6361.30 ± 913.68	8395.06 ± 5424.99	1.32	0.71	insig
dUMP	Nucleotide and its metabolomics	0.00 ± 0.00	12.67 ± 16.84	Inf	1.34	up
dAMP	Nucleotide and its metabolomics	156.87 ± 25.50	144.32 ± 15.18	0.92	0.89	insig
dCMP	Nucleotide and its metabolomics	0.00 ± 13.47	74.04 ± 9.30	1.18	1.13	insig
AMP	Nucleotide and its metabolomics	3343.78 ± 488.13	4102.18 ± 2209.07	1.23	0.64	insig
UDP-GlcNAc	Nucleotide and its metabolomics	1201.77 ± 966.86	2833.9 ± 2289.04	2.36	1.24	up
Cyclic-AMP	Nucleotide and its metabolomics	5.02 ± 1.69	5.92 ± 3.55	1.18	0.50	insig
Uracil	Nucleotide and its metabolomics	1743.71 ± 847.05	2861.50 ± 972.67	1.64	1.51	insig
Guanosine	Nucleotide and its metabolomics	708.36 ± 232.92	823.32 ± 78.59	1.16	0.89	insig
ADP	Nucleotide and its metabolomics	6444.74 ± 3128.65	16,236.92 ± 15,090.39	2.52	1.13	up
Nicotinamide adenine dinucleotide (NAD)	Nucleotide and its metabolomics	583.27 ± 249.29	680.24 ± 395.85	1.17	0.41	insig
Adenine	Nucleotide and its metabolomics	112.03 ± 23.70	189.67 ± 199.91	1.69	0.73	insig
Inosine	Nucleotide and its metabolomics	69,737.71 ± 16,818.32	72,341.10 ± 6477.25	1.04	0.31	insig
3-Phenyllactic-acid	Organic acid and its derivatives	12.31 ± 6.59	27.24 ± 15.66	2.21	1.60	up
Malic-acid	Organic acid and its derivatives	12,495.00 ± 5448.39	14,578.90 ± 5252.08	1.17	0.61	insig
Oxaloacetate	Organic acid and its derivatives	102.92 ± 148.03	444.60 ± 737.30	4.32	0.88	insig
Cis-aconitic-acid	Organic acid and its derivatives	68.59 ± 42.86	159.74 ± 103.59	2.33	1.52	up
Argininosuccinic acid	Organic acid and its derivatives	5259.25 ± 1049.08	6108.05 ± 857.80	1.16	1.23	insig
Itaconic acid	Organic acid and its derivatives	103.72 ± 56.72	218.61 ± 122.71	2.11	1.57	up
Fumaric acid	Organic acid and its derivatives	13,217.63 ± 6884.23	14,243.43 ± 6102.43	1.08	0.29	insig
Succinic acid	Organic acid and its derivatives	590.63 ± 496.18	365.73 ± 139.92	0.62	0.85	insig
Alpha-ketoglutaric acid	Organic acid and its derivatives	1665.55 ± 342.24	1462.18 ± 111.46	0.88	1.05	insig
Pyruvic-acid	Organic acid and its derivatives	3031.13 ± 900.63	2810.19 ± 438.87	0.93	0.46	insig
Citric-acid	Organic acid and its derivatives	2710.53 ± 1169.13	2999.77 ± 811.44	1.11	0.49	insig
Lactate	Organic acid and its derivatives	13,015.59 ± 2703.83	11,254.44 ± 2630.26	0.86	0.89	insig
d-Erythrose-4-phosphate	Phosphate sugars	11,810.77 ± 1626.72	11,475.31 ± 1666.78	0.97	0.24	insig
Trehalose-6-phosphate	Phosphate sugars	8.15 ± 1.42	12.35 ± 2.51	1.52	1.87	insig
d(+)-Glucose	Phosphate sugars	12,290.30 ± 4122.4	4928.22 ± 3589.92	0.40	1.96	down
6-Phosphogluconic-acid	Phosphate sugars	4128.68 ± 1513.17	4456.16 ± 2958.2	1.08	0.20	insig
d-Fructose-6-phosphate	Phosphate sugars	648.87 ± 597.40	516.79 ± 577.37	0.80	0.26	insig
d-Glucose-6-phosphate	Phosphate sugars	3851.37 ± 2379.94	2952.62 ± 2106.99	0.77	0.53	insig
d-Ribulose-5-phosphate	Phosphate sugars	61,620.10 ± 24,922.22	85,522.10 ± 17,042.26	1.39	1.40	insig
Fructose-1,6-bisphosphate	Phosphate sugars	288.49 ± 171.66	566.16 ± 457.50	1.96	1.09	insig
d-Glucose-1-phosphate	Phosphate sugars	547.00 ± 602.17	500.64 ± 653.22	0.92	0.04	insig
Sedoheptulose-7-phosphate	Phosphate sugars	55.32 ± 123.70	220.73 ± 123.55	3.99	1.63	up
Glycerol-3-phosphate	Phosphoric acids	1147.22 ± 507.86	1799.95 ± 1499.94	1.57	0.82	insig
Phosphoenolpyruvic-acid	Phosphoric acids	1.27 ± 2.84	0.00 ± 0.00	0.00	0.91	insig
3-Phosphoglycerate	Phosphoric acids	188.54 ± 41.34	194.51 ± 23.49	1.03	0.27	insig
Dihydroxyacetone-phosphate	Phosphoric acids	1617.99 ± 246.94	2340.89 ± 808.33	1.45	1.47	insig
Glyceraldehyde-3-phosphate	Phosphoric acids	1908.03 ± 306.56	2766.23 ± 1017.19	1.45	1.43	insig
2-Phospho-d-glyceric acid	Phosphoric acids	199.19 ± 53.26	195.08 ± 32.46	0.98	0.12	insig

The VIP value was based on OPLS-DA model (biological repetition ≥ 3). The differential metabolites were screened by the fold change and VIP value: select metabolites with VIP > 1; Select metabolites with fold change ≥ 2 or fold change ≤ 0.5. “Type” indicated the changes in differential metabolites; “insig” indicated the metabolite was not different between groups; “up” indicated up-regulation; “down” indicated down-regulation

### The changes in metabolites in the quadriceps muscle were associated with the activity of the AMPK/SIRT1/PGC1α pathway induced by GqDNVs

To investigate the effects of GqDNVs on muscle atrophy mice, we analyzed the relationships between energy-related metabolites and other indicators of muscle health in the DEX group and DG group through the Spearman rank analysis (Fig. 8c). The cross-sectional area of the quadriceps muscle was positively correlated with serum LDH and negatively associated with glucose in the quadriceps muscle. The grip strength of mice was positively correlated with dUMP, UDP-GlcNAc, cis-aconitic-acid, itaconic-acid, sedoheptulose-7-phosphate and especially ADP in the quadriceps muscle. The grip strength was negatively correlated with SOD in muscle. The running distance was negatively correlated with CK in muscles, and stride length was positively related to flavin-mononucleotide and 3-phenyllactic-acid.

Moreover, the expression levels of proteins in signal pathways were closely associated with the function and quality of skeletal muscle. The weight, cross-sectional area of the quadriceps muscle and stride length were positively correlated to the p-AMPK/AMPK. The protein levels of PGC1α, SIRT3 and MYOG were positively associated with the cross-sectional area of quadriceps muscle and stride length. Notably, there were prominent associations between the proteins of signal pathways and energy-related metabolites in the quadriceps muscle, especially the relationships between the differential metabolites and SIRT1.

## Discussion

Goji contains many highly biologically active nutrients, such as flavonoids, polysaccharides, carotenoids, anthocyanins, phenolic acids, and alkaloids [32, 33]. *Lycium barbarum* polysaccharides (LBPs), the main active components of goji, are polysaccharides with molecular weight in the range of 10–2300 kDa [34] and are 5–8% of the total dry matter of goji [35]. Since the monosaccharide composition and molecular structure of LBPs are based on the biodiversity of goji (genomic diversity, geographical origin and environmental conditions), the compositions of LBPs were reported dissimilarly in different research [36]. In general, LBPs are considered to be composed of six monosaccharides (galactose, glucose, rhamnose, arabinose, mannose and xylose) and antioxidants [37, 38].

In this study, we isolated exosome-like nanoparticles (GqDNVs) from fresh goji (berry of *Lycium barbarum* L.). Meanwhile, we found that GqDNVs have similar components to goji [32]. Saccharides (35.54%) accounted for the majority proportion, and the components are closely associated with the metabolism-related pathways through the non-targeted metabolome analysis and KEGG annotations. Therefore, we further conducted targeted metabolome analysis of saccharides in GqDNVs since LBPs were the main active component of goji. Our results also showed that the saccharides of GqDNVs were similar to LBPs, including glucose, fructose, maltose, mannose, rhamnose and xylose.

Referring to previous reports for exosomes derived from animals and other sources [39, 40], GqDNVs could also be absorbed into the C2C12 cells directly without apparent toxicity on cell viability. Notably, GqDNVs were mainly clustered around the nucleus. Our images of live mice demonstrated that GqDNVs were primarily distributed in the left thigh of mice after being injected into the left quadriceps muscle. After the muscles were dissected, fluorescence was predominantly found in the left quadriceps muscle, and fluorescence in other muscles was not apparent. These pictures indicated that GqDNVs could be maintained for a long time and act as a sustained-release function in muscles. Therefore, GqDNVs not only exert their pharmacological or nutrient functions but also make a probable vector for fighting muscle aging or curing muscle diseases in the future.

Dexamethasone for mimicking muscle aging or muscle atrophy model has been reported in various studies [41, 42]. In this study, we found that GqDNVs increased the grip strength and the cross-sectional area of the quadriceps muscle in the DG group after the administration of dexamethasone, which was consistent with the effect of goji [11, 43]. Similar alterations were also shown in the C2C12 cells. Meng et al. showed similar results that the extract of goji could regulate skeletal muscle remodeling in a manner similar to exercise, enhance muscle endurance by up-regulating aerobic respiration, significantly increase the mass of the tibialis anterior muscle and the gastrocnemius muscle of mice and improve the average running distance of mice [11]. However, the weight of skeletal muscle in this study, including the quadriceps muscle, did not change after GqDNVs were administrated in the DG group compared with the DEX group. The non-significant effect of GqDNVs on improving the weight of skeletal muscle in muscle atrophy mice might be because of the period (14 days) of the intervention in our study, which was shorter than the intervention (180 days) in the previous report [11]. Therefore, we hold the opinion that GqDNVs can exert a biologically active effect similar to goji, which could also improve the grip strength and the cross-sectional area of the quadriceps muscle. Besides, we administrated mice with GqDNVs through injection instead of oral in this study, which could directly contact target organs and quickly be absorbed and utilized by cells. The GqDNVs improved grip strength and the cross-sectional area of the quadriceps muscle in muscle atrophy mice with short-term intervention (14 days), demonstrating the potential high bioavailability of GqDNVs.

The level of serum LDH was increased in the DG group compared with the DEX group. Notably, the cross-sectional area of the quadriceps muscle was positively correlated to the activity of serum LDH through the Spearman rank analysis. Consistently, LBPs could significantly increase the muscle glycogen and liver glycogen reserves of mice and increase the serum LDH activity [44]. Higher serum LDH activity was connected to high-intensity training that can induce an antioxidative enzyme synthesis path [45]. However, the activities of LDH, MDA, and CK in the gastrocnemius muscle were not changed by GqDNVs in this study. Meantime, LBPs were found to reduce the level of oxidative stress in the skeletal muscle of subhealthy mice [46] and rats with exhaustive motor induction [47]. Another study also demonstrated a similar result in which the intervention period of goji in rabbits (56 days) [48] was longer than the period in our study (14 days). We supposed that the differences might be attributed to the state and administration ways or times of the experimental animal. The mice and rats in previous research were in a state of exhaustion or fatigue, but our mice were interved with dexamethasone.

The results of the non-targeted metabolome analysis showed that the contents of GqDNVs had close associations with metabolism. In muscle atrophy mice induced by dexamethasone, GqDNVs improved the grip strength and the cross-sectional area of quadriceps muscle fibers. Thus, we preferred to choose the signal proteins related to muscle growth and metabolism to explore the molecular mechanism of the effect of GqDNVs. In the current studies, AMPK, PGC1α, SIRT1 and SIRT3 have been considered to affect skeletal muscle metabolism. AMPK is a central regulator of metabolic homeostasis, especially in skeletal muscle cells, which controls the growth and size of muscle fibers [49, 50]. AMPK can cooperate with PGC1α, SIRT1 and SIRT3 to increase mitochondrial protein synthesis, while AMPK activation can inhibit the mTOR pathway’s activity [51]. SIRT1 can enhance the expression of PGC1α, and MYOD acts as a positive intermedia in this process [52]. SIRT1 is mainly localized in the nucleus and SIRT3 is present in the mitochondria, which regulate lipids and glucose metabolism and act as crucial regulators of energy homeostasis [53]. Meanwhile, in the previous studies on goji berries and their extract, LBPs could reduce the level of oxidative stress in the skeletal muscle of rats [46, 47] and the extract of goji and betaine increased the expression of phosphorylation of AMPK, PGC1α and SIRT1 in C2C12 cells [12]. Therefore, we focused on the expression levels of AMPK and its downstream proteins in this study. Our present results documented that GqDNVs improved the activities of AMPK, PGC1α, SIRT1 and SIRT3, which is consistent with the KEGG pathways of metabolites in GqDNVs [pathways in metabolism, including biosynthesis of amino acids (ko01230) and biosynthesis of cofactors (ko01240)].

In muscle atrophy model mice induced by dexamethasone, GqDNVs also increased expression levels of myogenic regulatory factors (MYF5, MYOD and MYOG). Myogenic regulatory factors MYF5, MYOD and MYOG are transcription factors that are highly conserved and essential for skeletal myogenesis, muscle proliferation and muscle differentiation [54–56]. The up-regulated protein and mRNA expression levels of MYF5, MYOD and MYOG in muscle atrophy model mice demonstrated that GqDNVs activated muscle regeneration in our study. Moreover, we found increases in the mRNA expression level of MYH7 in the quadriceps muscle and decreases in MYH2 and MYH4 in the tibialis anterior muscle after dexamethasone administration. GqDNVs could significantly reduce the mRNA expression level of MYH7 in quadriceps muscle, while the mRNA expression levels of MYH2 and MYH4 in quadriceps and tibialis anterior muscle had upward trends insignificantly. It was indicated that dexamethasone induced the fast to slow transition of myosin heavy chain isoform in muscle [52], and GqDNVs inhibited the transition.

As for testifying above results, when inhibiting AMPK in C2C12 cells, we found apparent decreases in AMPK, PGC1α, SIRT1 and MYOG activity. However, PGC1α inhibition did not affect the activity of SIRT1 and MYOG in C2C12 cells, while the increased expression level of p-AMPK might be induced by a compensatory reaction. Therefore, the AMPK pathway played a significant role in improving skeletal muscle caused by GqDNVs. The PGC1α also took part in this effect of GqDNVs but was not the critical protein. The close relationships between cross-sectional area, grip strength and protein levels based on the Spearman rank analysis supported this hypothesis. The alternations induced by GqDNVs in skeletal muscle might be closely associated with mitochondria and metabolic pathways.

To further explore the changes in skeletal muscle caused by GqDNVs, we compared the differences in energy-related metabolites in quadriceps muscle between the DEX and DG groups through the targeted metabolome analysis. Based on the KEGG database, the differential metabolites in skeletal muscle showed the up-regulated metabolic pathways. Therefore, we used the Spearman rank analysis to investigate and testify the relationships between energy-related metabolites in quadriceps muscle and the quality and function of skeletal muscle. We combined the results of energy-related metabolome analysis in quadriceps muscle to explore the relationship between metabolism, signaling pathways and skeletal muscle health indicators. The activated biosynthesis of nucleotide sugars (k001250) and amino sugar and nucleotide sugar metabolism (k000520), induced by up-regulated UDP-GlcNAc and sedoheptulose-7-phosphate and down-regulated glucose, were related to the activation of SIRT1 induced by GqDNVs. The pathways of autophagy (k004140), endocytosis (k004144) and lysosome (k004142) were activated by up-regulated ADP and guanosine-diphosphate in the DG group compared with the DEX group, which was associated with SIRT1. It was indicated in this study that autophagy was activated in the quadriceps muscle, which implicated the promotion of muscle regeneration in sarcopenia [57]. Moreover, the up-regulated flavin-mononucleotide and ADP, involved in oxidative phosphorylation (k000190), were related to the activation of AMPK and SIRT1 induced by GqDNVs. These results indicated that SIRT1 played a critical role in the effect of GqDNVs on skeletal muscle. SIRT1 was involved in improving mitochondrial function [58] and anti-skeletal muscle atrophy [59], which enhanced the repair process after muscle injury and actively participated in muscle hypertrophy through up-regulation of anabolic and down-regulation of catabolic processes [60]. Furthermore, the level of ATP in quadriceps muscle was not different between the DEX and DG groups, though the level of ATP showed an increased pattern induced by GqDNVs. Notably, GqDNVs increased the level of ATP and alleviated the damage in mitochondria in C2C12 cells with the administration of dexamethasone, which side confirmed that GqDNVs might activate the oxidative phosphorylation process [61]. This inconsistency might be due to the more significant individual differences in mice than in C2C12 cells. Therefore, the positive effects of GqDNVs on skeletal muscle were associated with the activated oxidative phosphorylation pathway related to mitochondrial function.

In summary, this study found that GqDNVs can improve muscle regeneration in muscle atrophy mice through the AMPK/SIRT1/PGC1α pathway, chiefly attributed to the activation of AMPK. However, there are still some limit points in our study. We have focused on the saccharides of GqDNVs because the LBPs are the main active components of *Lycium barbarum* L. The other contents of GqDNVs and their effects need further investigation. The impacts of GqDNVs and their contents, especially the LBPs, also need to be compared. Besides, in our study, the injection of GqDNVs can be directly absorbed in the quadriceps muscle except for the first-pass effect. It is necessary to explore the systemic action of GqDNVs with oral treatment closer to ordinary behavior.

## Conclusion

GqDNVs are derived from the fresh berry of *Lycium barbarum* L. (goji) and are rich in saccharides and lipids. Based on the KEGG pathways of annotated metabolites in non-targeted metabolome analysis, GqDNVs are associated with metabolism-related pathways. GqDNVs can improve the cross-sectional area of quadriceps muscle and grip strength in muscle atrophy model mice and activate the AMPK/SIRT1/PGC1α pathway in which the activation of AMPK played the chief role. In the results of energy-targeted metabolome analysis in the quadriceps muscle, we find that the changed metabolic pathways were associated with the activated oxidative phosphorylation pathway. Therefore, GqDNVs improve muscle regeneration in muscle atrophy mice and have the potential as an anti-skeletal muscle aging agent, while their effects on other organs and mechanisms need further study.

### Supplementary Information

Supplementary Material 1.

Supplementary Material 2.

Supplementary Material 3.

## Data Availability

All relative data are included in this article and its supplementary information files.

## Abbreviations

ATP: Adenosine triphosphate
ABC transporter: ATP-binding cassette transporter
ADP: Adenosine diphosphate
AMPK: Activating AMP-activated protein kinase
ANOVA: Analysis of variance
CCK-8: Cell counting kit-8
CK: Creatine kinase
DA score: Differential abundance score
DAPI: 4′,6-Diamidino-2-phenylindole
DMEM: Dulbecco’s modified Eagle’s medium
dUMP: Deoxyuridine monophosphate
FC: Fold change
FITC: Fluorescein isothiocyanate
GqDNVs: Gouqi-derived nanovesicles
JC-1: 5,5′,6,6′-Tetrachloro-1,1′,3,3′-tetraethyl-imidacarbocyanine iodide
KEGG: Kyoto Encyclopedia of Genes and Genomes
LDH: Lactate dehydrogenase
LBPs: *Lycium barbarum* polysaccharides
MDA: Malondialdehyde
MYF5: Myogenic factor 5
MYH2: Myosin heavy chain 2
MYH4: Myosin heavy chain 4
MYH7: Myosin heavy chain 7
MYOD: Myogenic differentiation
MYOG: Myogenin
NTA: Nanoparticle tracking analysis
OPLS-DA: Orthogonal partial least squares-discriminant analysis
PGC1α: Peroxisome proliferator-activated receptor gamma coactivator1-alpha
PBS: Phosphate buffer saline
PCA: Principal component analysis
Q-PCR: Quantitative real-time polymerase chain reaction
ROI: Region of area
SD: Standard deviation
SE: Standard error
SIRT1: Sirtuin 1
SIRT3: Sirtuin 3
SOD: Superoxide dismutase
UDP-GlcNAc: Uridine diphosphate *N*-acetylglucosamine orchestrates
VIP: Variable importance in projection
