# Supplementary material for: Gouqi-derived nanovesicles (GqDNVs) inhibited dexamethasone-induced muscle atrophy associating with AMPK/SIRT1/PGC1α signaling pathway
Source: J Nanobiotechnology. 2024 May 22;22:276. doi: 10.1186/s12951-024-02563-9 (PMC11112783; doi:10.1186/s12951-024-02563-9)

**Gouqi-derived Nanovesicles (GqDNVs) Inhibited Dexamethasone-induced Muscle Atrophy Associating with AMPK/SIRT1/PGC1α Signaling Pathway**

Xiaolei Zhou^1,2^, Shiyin Xu^1,2^, Zixuan Zhang^1,2^, Mingmeng Tang^1,2^, Zitong Meng^1,2^, Zhao Peng^1,2^, Yuxiao Liao^1,2^, Xuefeng Yang^1,2^, Andreas K. Nüssler^3^, Liegang Liu^1,2^, Wei Yang^1,2^*

1. Department of Nutrition and Food Hygiene, Hubei Key Laboratory of Food Nutrition and Safety, Tongji Medical College, Huazhong University of Science and Technology, Hangkong Road 13, 430030, Wuhan, China.

2. Department of Nutrition and Food Hygiene and MOE Key Lab of Environment and Health, School of Public Health, Tongji Medical College, Huazhong University of Science and Technology, Hangkong Road 13, 430030, Wuhan, China.

3. Department of Traumatology, BG Trauma Center, University of Tübingen, Schnarrenbergstr. 95, 72076 Tübingen, Germany.

*: Dr. Wei Yang is the corresponding author and to whom correspondence should be addressed:

Dr. Wei Yang, PhD, Associate Professor, Department of Nutrition and Food Hygiene and MOE Key Lab of Environment and Health, School of Public Health, Tongji Medical College, Huazhong University of Science and Technology, 13 Hangkong Road, Wuhan, 430030, China Tel.: +86 27 83650522; Fax: +86 27 83650522, E-mail address: [yw8278@hotmail.com](mailto:yw8278@hotmail.com) or yw8278@hust.edu.cn

**Fig S1.** The representative of the quantity of fluorescence-positive C2C12 cells treated with PKH 26 labeled-GqDNVs through fluorescence-activated cell sorting. a: The C2C12 cells were treated with PBS (Blank group). b: The C2C12 cells were labeled with PKH 26 (Positive group). c: The C2C12 cells were treated with the PKH 26-labeled GqDNVs (GqDNVs group). a-c: The number on the ordinate (Events) indicates the number of cells. The horizontal coordinate shows the fluorescence intensity. The percentage on the graph indicates the percentage of C2C12 cells that are fluorescent-positive to the total number of cells.


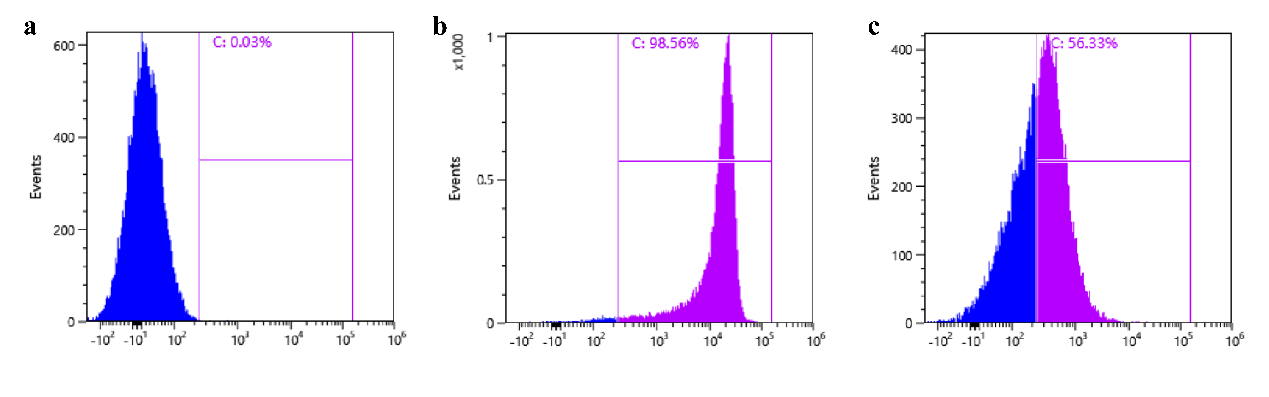


**Fig S2.** The mitochondrial membrane potential of the C2C12 cells was detected by JC-1. JC-1 accumulates in the matrix of mitochondria with high membrane potential and forms aggregates, which produce red fluorescence. JC-1 is a monomer in the matrix of the mitochondria with low membrane potential, which can produce green fluorescence. The area of JC-1 monomers and aggregates was measured (mean ± SD). One-way ANOVA analysis and Tukey’s multiple comparison analysis were used in the between-group comparisons. “*”, “**”, “***” and “****” indicate that after Tukey’s multiple comparison analysis, the *P*-value is lower than 0.05, 0.01, 0.001 and 0.0001.


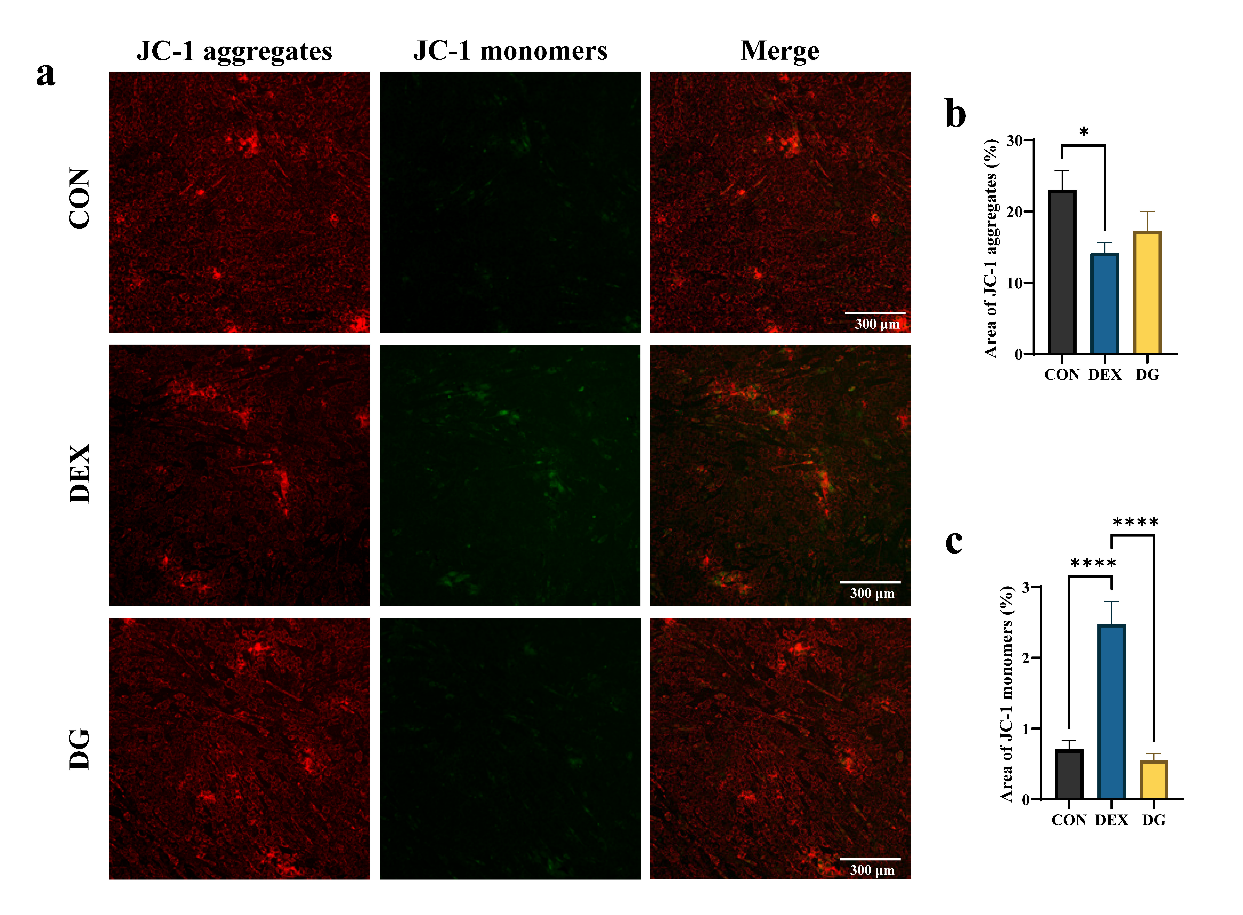


**Fig S3.** The body weight of mice after 9 days of 25 mg/kg·d dexamethasone intervention (mean ± SD). One-way ANOVA analysis and Tukey’s multiple comparison analysis were used in the between-group comparisons. The ANOVA analysis reports no differences between groups. “ns” indicates that after Tukey’s multiple comparison analysis, the *P*-value is higher than 0.05.

**Fig S4.** The grip strength of mice after 9 days of 25 mg/kg·d dexamethasone intervention (mean ± SD). One-way ANOVA analysis and Tukey’s multiple comparison analysis were used in the between-group comparisons. “ns” indicates that after Tukey’s multiple comparison analysis, the *P*-value is higher than 0.05. “*”, “**”, “***” and “****” indicate that after Tukey’s multiple comparison analysis, the *P*-value is lower than 0.05, 0.01, 0.001 and 0.0001.

**Fig S5.** The running distance of mice after 9 days of 25 mg/kg·d dexamethasone intervention (mean ± SD). One-way ANOVA analysis and Tukey’s multiple comparison analysis were used in the between-group comparisons. “ns” indicates that after Tukey’s multiple comparison analysis, the *P*-value is higher than 0.05. “*”, “**”, “***” and “****” indicate that after Tukey’s multiple comparison analysis, the *P*-value is lower than 0.05, 0.01, 0.001 and 0.0001.

**Fig S6.** The activities of CK in muscle after the 24-day intervention (mean ± SD). One-way ANOVA analysis and Tukey’s multiple comparison analysis were used in the between-group comparisons. The ANOVA analysis reports no differences between groups. “ns” indicates that after Tukey’s multiple comparison analysis, the *P*-value is higher than 0.05.

**Fig S7.** The levels of LDH in muscle after the 24-day intervention (mean ± SD). One-way ANOVA analysis and Tukey’s multiple comparison analysis were used in the between-group comparisons. The ANOVA analysis reports no differences between groups. “ns” indicates that after Tukey’s multiple comparison analysis, the *P*-value is higher than 0.05.

**Fig S8.** The levels of MDA in muscle after the 24-day intervention (mean ± SD). One-way ANOVA analysis and Tukey’s multiple comparison analysis were used in the between-group comparisons. The ANOVA analysis reports no differences between groups. “ns” indicates that after Tukey’s multiple comparison analysis, the *P*-value is higher than 0.05.

**Fig S9.** The activities of SOD in muscle after the 24-day intervention (mean ± SD). One-way ANOVA analysis and Tukey’s multiple comparison analysis were used in the between-group comparisons. The ANOVA analysis reports no differences between groups. “ns” indicates that after Tukey’s multiple comparison analysis, the *P*-value is higher than 0.05.

**Fig S10.** The quadriceps muscle and the ratio of quadriceps muscle weight and body weight of mice after the 24-day intervention (mean ± SD). a: The weight of left and right quadriceps muscle in mice after being dissected. b: The ratio of quadriceps muscle weight and body weight in mice. One-way ANOVA analysis and Tukey’s multiple comparison analysis were used in the between-group comparisons. The ANOVA analysis reports no differences between groups. “ns” indicates that after Tukey’s multiple comparison analysis, the *P*-value is higher than 0.05.


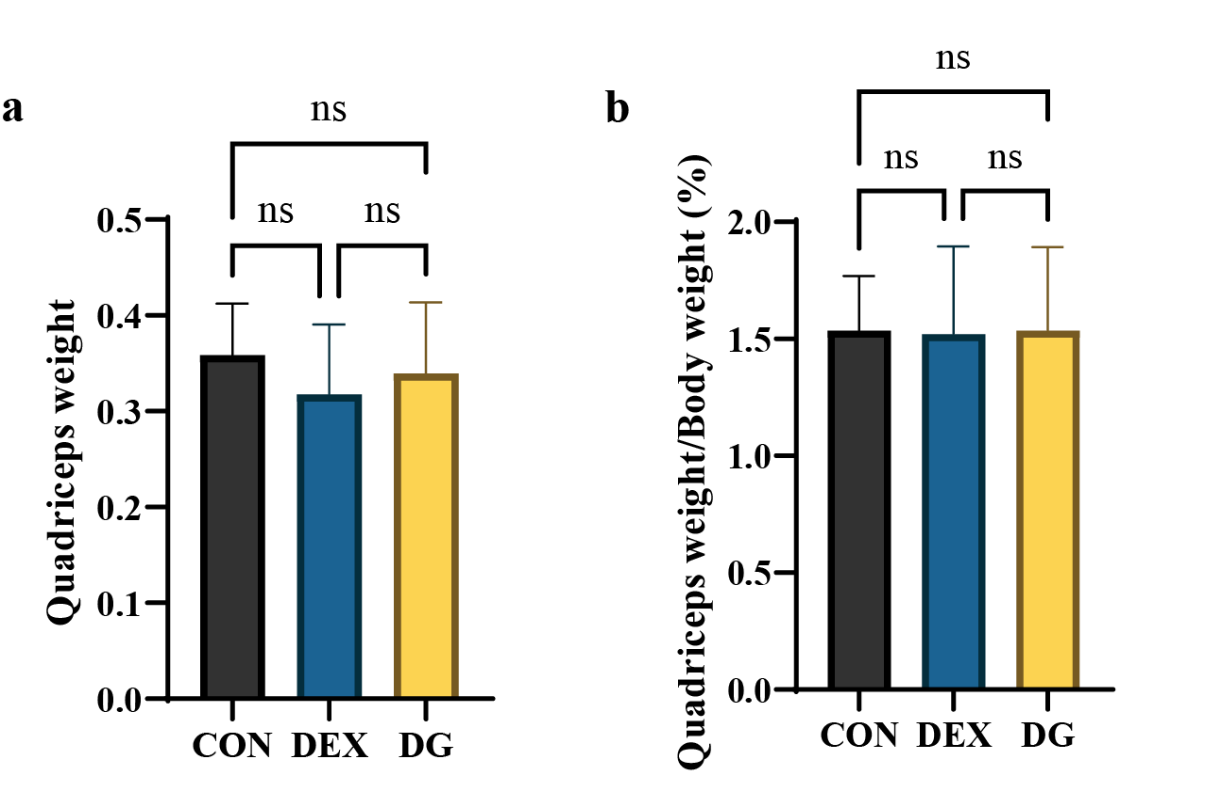


**Fig S11.** The stride length of mice after the 24-day intervention (mean ± SD). a: The image of mice walking imprinting. The hind legs were marked with blue ink. The forelimbs were marked with red ink. b: The average stride length in the 3 groups. One-way ANOVA analysis and Tukey’s multiple comparison analysis were used in the between-group comparisons. The ANOVA analysis reports no differences between groups. “ns” indicates that after Tukey’s multiple comparison analysis, the *P*-value is higher than 0.05.


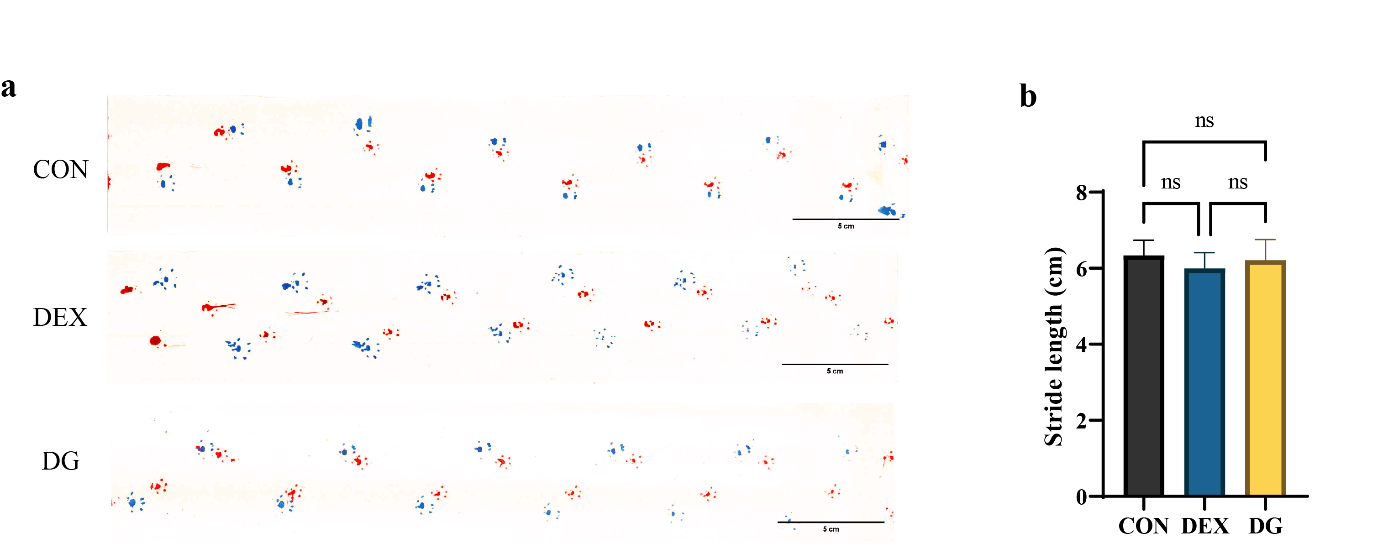


**Fig S12.** The changes of mRNA expression levels induced by GqDNVs and dexamethasone in quadriceps muscle of mice through Q-PCR. a-f: The quantitative analyses of AMPK, SIRT1, PGC1α, MYF5, MYOG and MYOD gene expression levels (mean ± standard error (SE)). All the mRNA expression levels were normalized to the GAPDH. N = 6 in each group. One-way ANOVA and Tukey’s multiple comparison analysis were used in the between-group comparisons. “*”, “**”, “***” and “****” indicate that after Tukey’s multiple comparison analysis, the *P*-value is lower than 0.05, 0.01, 0.001 and 0.0001.


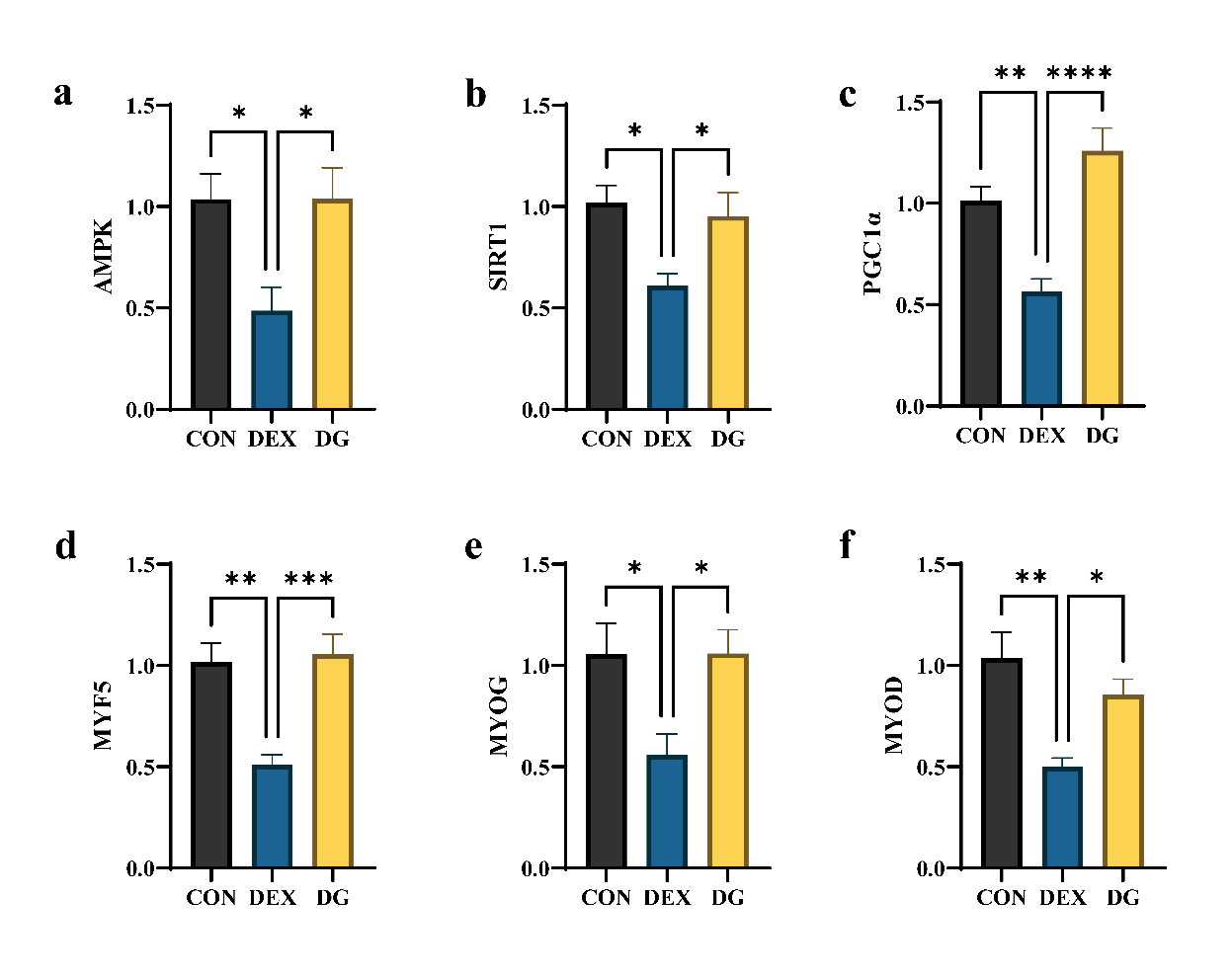


**Fig S13.** The changes of mRNA expression levels of myosin heavy chain isoforms induced by GqDNVs and dexamethasone in quadriceps muscle of mice through Q-PCR. a-f: The quantitative analyses of MYH2, MYH4 and MYH7 gene expression levels (mean ± SE). All the mRNA expression levels were normalized to the GAPDH. N = 6 in each group. One-way ANOVA and Tukey’s multiple comparison analysis were used in the between-group comparisons. “*”, “**”, “***” and “****” indicate that after Tukey’s multiple comparison analysis, the *P*-value is lower than 0.05, 0.01, 0.001 and 0.0001.


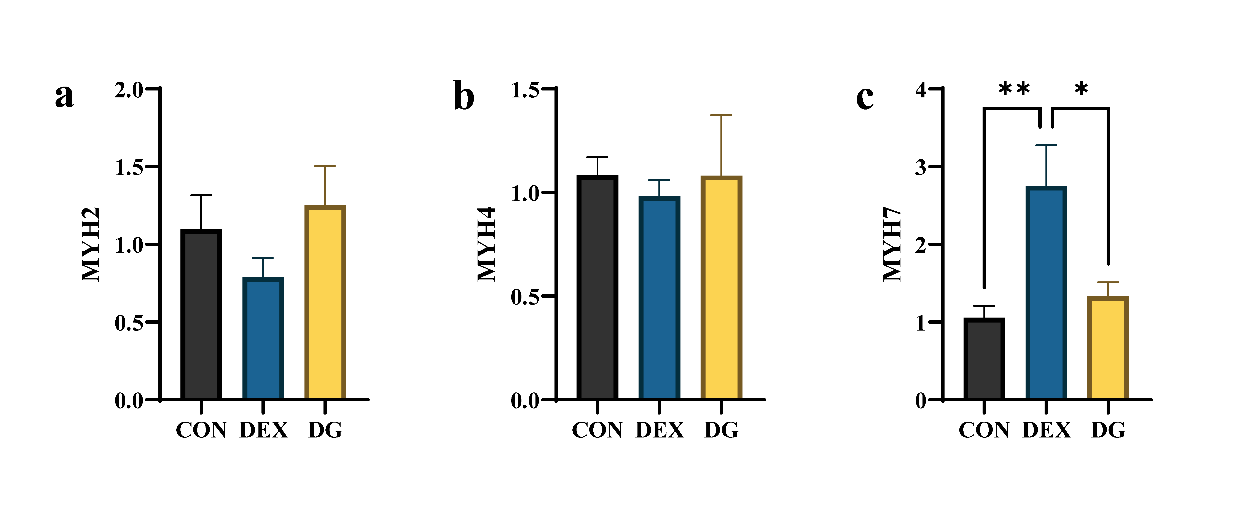


**Fig S14.** The changes of mRNA expression levels of myosin heavy chain isoforms induced by GqDNVs and dexamethasone in tibialis anterior muscle of mice through Q-PCR. a-f: The quantitative analyses of MYH2, MYH4 and MYH7 gene expression levels (mean ± SE). All the mRNA expression levels were normalized to the GAPDH. N = 6 in each group. One-way ANOVA and Tukey’s multiple comparison analysis were used in the between-group comparisons. “*”, “**”, “***” and “****” indicate that after Tukey’s multiple comparison analysis, the *P*-value is lower than 0.05, 0.01, 0.001 and 0.0001.


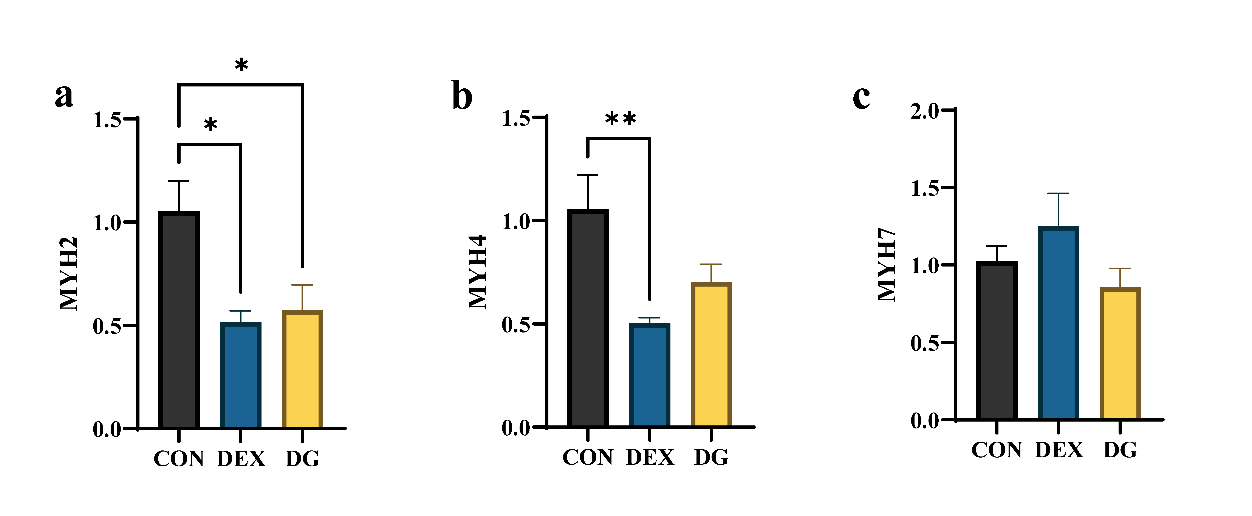

Supplement: Supplementary file 2 — Supplementary Material 2. [file 12951_2024_2563_MOESM2_ESM.docx]
