# Supplementary material for: Gouqi-derived nanovesicles (GqDNVs) inhibited dexamethasone-induced muscle atrophy associating with AMPK/SIRT1/PGC1α signaling pathway
Source: J Nanobiotechnology. 2024 May 22;22:276. doi: 10.1186/s12951-024-02563-9 (PMC11112783; doi:10.1186/s12951-024-02563-9)
Supplement: Supplementary file 3 — Supplementary Material 3. [file 12951_2024_2563_MOESM3_ESM.docx]

**Gouqi-derived Nanovesicles (GqDNVs) Inhibited Dexamethasone-induced Muscle Atrophy Associating with AMPK/SIRT1/PGC1α Signaling Pathway**

Xiaolei Zhou^1,2^, Shiyin Xu^1,2^, Zixuan Zhang^1,2^, Mingmeng Tang^1,2^, Zitong Meng^1,2^, Zhao Peng^1,2^, Yuxiao Liao^1,2^, Xuefeng Yang^1,2^, Andreas K. Nüssler^3^, Liegang Liu^1,2^, Wei Yang^1,2^*

1. Department of Nutrition and Food Hygiene, Hubei Key Laboratory of Food Nutrition and Safety, Tongji Medical College, Huazhong University of Science and Technology, Hangkong Road 13, 430030, Wuhan, China.

2. Department of Nutrition and Food Hygiene and MOE Key Lab of Environment and Health, School of Public Health, Tongji Medical College, Huazhong University of Science and Technology, Hangkong Road 13, 430030, Wuhan, China.

3. Department of Traumatology, BG Trauma Center, University of Tübingen, Schnarrenbergstr. 95, 72076 Tübingen, Germany.

*: Dr. Wei Yang is the corresponding author and to whom correspondence should be addressed:

Dr. Wei Yang, PhD, Associate Professor, Department of Nutrition and Food Hygiene and MOE Key Lab of Environment and Health, School of Public Health, Tongji Medical College, Huazhong University of Science and Technology, 13 Hangkong Road, Wuhan, 430030, China Tel.: +86 27 83650522; Fax: +86 27 83650522, E-mail address: [yw8278@hotmail.com](mailto:yw8278@hotmail.com) or yw8278@hust.edu.cn

1. **Footprint analysis**

Footprint analysis was performed to assess gait and motor coordination on day 24. The front and hind paws of mice were stained with red and blue dyes, respectively. The mice were then encouraged to walk straight on a paper-lined runway. The stride lengths (hind paws (blue)) were measured and analyzed only when the mice ran at a constant velocity (1).

1. **Relative indicators of oxidative stress and muscle function**

The samples of gastrocnemius muscle were collected and stored at -80 °C. The amount of lactate dehydrogenase (LDH) and creatine kinase (CK) in gastrocnemius muscles was determined using commercial kits (Nanjing JianCheng Bioengineering Institute, China). The concentrations of malondialdehyde (MDA) and superoxide dismutase (SOD) were determined using commercial kits (Beyotime, China). The absorbance values were measured with a microplate reader (Infinite M Nano, Tecan, Switzerland).

1. **Metabolomics analysis**
   1. **Non-targeted metabolome analysis about GqDNVs**

Take out the sample of GqDNVs (1×10^10^ particles/mL, 1 mL) from the -80 °C refrigerator and thaw it on ice. Add 500 μL of 80% methanol internal standard extract precool at -20 °C and vortex for 2 min. Put the sample tube into liquid nitrogen for quickly freezing for 5 min, then take it out and thaw it on ice for 5 min, vortex for 2 min, and repeat this operation three times. Centrifuge at 12000 r/min for 10 min at 4 °C, pipette the supernatant and concentrate until completely dry. Added 1200 μL of 80% methanol extract, vortex for 3 min, and ice water bath ultrasound for 15 min. Centrifuged at 12000 r/min for 10 min at 4 °C, pipette 200 μL of the supernatant, and passed the protein precipitation plate for LC/MS analysis.

The sample extracts were analyzed using the UPLC-ESI-MS/MS system (UPLC, ExionLC™ AD,https://sciex.com.cn/; MS, Applied Biosystems 6500 Q TRAP, <https://sciex.com.cn/>). The analytical conditions were as follows: UPLC: column, Agilent SB-C18 (1.8 µm, 2.1 mm × 100 mm); The mobile phase consisted of solvent A, pure water with 0.1% formic acid, and solvent B, acetonitrile with 0.1% formic acid. Sample measurements were performed with a gradient program that employed the starting conditions of 95% A, 5% B. Within 9 min, a linear gradient to 5% A, 95% B was programmed, and a composition of 5% A, 95% B was kept for 1 min. Subsequently, a composition of 95% A and 5.0% B was adjusted within 1.1 min and held for 2.9 min. The flow velocity was 0.35 mL per minute; The column oven was set to 40 °C; The injection volume was two μL. The effluent was alternatively connected to an ESI-triple quadrupole-linear ion trap (QTRAP)-MS. The ESI source operation parameters were as follows: source temperature 500 °C; ion spray voltage 5500 V (positive ion mode)/-4500 V (negative ion mode); ion source gas I, gas II, and curtain gas were set at 50, 60, and 25 psi, respectively; the collision-activated dissociation was high. Triple quadrupole scans were acquired as MRM experiments with collision gas (nitrogen) set to medium. Declustering potential and collision energy for individual MRM transitions were done with further declustering potential and collision energy optimization. A specific set of MRM transitions was monitored for each period according to the metabolites eluted within this period (2, 3).

- - 1. **Saccharides-targeted metabolome analysis about GqDNVs**

Methanol was purchased from Merck (Darmstadt, Germany). MilliQ water (Millipore, Bradford, USA) was used in all experiments. All of the standards were purchased from CNW (Shanghai), IsoReag (Shanghai) and TCI (Shanghai). The stock solutions of standards were prepared at the concentration of 2 mg/mL in methanol. All stock solutions were stored at -20 °C. The stock solutions were diluted with methanol to working solutions before analysis.

The sample of GqDNVs (1×10^10^ particles/mL, 1 mL) was removed from the -80°C refrigerator and thawed on ice. The 500 μL GqDNVs samples were concentrated to dry and 100 μL of ultrapure water extract (containing protease inhibitors, PMSF and EDTA) was added to resuspend the cell pellet. The mixture was freeze-thawed in liquid nitrogen and repeated three times. The 50 μL of the suspension was transferred into a new tube with 500 μL of methanol: isopropanol: water (3:3:2, v/v/v) solution. The sample tube was vortexed for 3 min and ultrasonicated for 30 min. After that, the extract was centrifuged at 12,000 rpm under 4 °C for 3 min. The supernatant (200 μL) was mixed with 20 μL internal standard (250 μg/mL) and evaporated under a nitrogen gas stream. The vaporized sample was transferred to the lyophilizer for freeze-drying. The residue was used for the further derivatization. The left cell suspension was used to analyze the total protein with the BCA Protein Assay kit. The derivatization method was as follows: the sample was mixed with a 100 μL solution of methoxyamine hydrochloride in pyridine (15 mg/mL). The mixture was incubated at 37 °C for 2 h. Then 100 μL of BSTFA was added into the mix and kept at 37 °C for 30 min after vortex-mixing. GC-MS analyzed the mixture after diluting to an appropriate concentration (4-6).

Agilent 8890 gas chromatograph coupled to a 5977B mass spectrometer with a DB-5MS column (30 m length × 0.25 mm i.d. × 0.25 μm film thickness, J&W Scientific, USA) was employed for GC-MS analysis of sugars. Helium was used as carrier gas at a 1 mL/min flow rate. Injections were made in the split mode with a split ratio 5:1 and the injection volume was 1 μL. The oven temperature was held at 160 °C for 1 min and then raised to 200 °C at a speed of 6 °C/min, raised to 270 °C at 10 °C/min, raised to 300 °C at 5 °C/min, raised to 320 °C at 20 °C/min and held at the temperature for 5.5 min. All samples were analyzed in selective ion monitoring mode. The ion source and transfer line temperature were 230 °C and 280 °C, respectively (4, 7).

- 1. **Metabolome analysis of muscle samples of mice after GqDNVs treatment**

HPLC-grade acetonitrile and methanol were purchased from Merck (Darmstadt, Germany). MilliQ water (Millipore, Bradford, USA) was used in all experiments. The standards were purchased from Sigma-Aldrich (St. Louis, MO, USA). Formic acid was bought from Sigma-Aldrich (St. Louis, MO, USA). The stock solutions of standards were prepared at the concentration of 1 mg/mL in methanol and other solutions. All stock solutions were stored at -20 °C. The stock solutions were diluted with methanol to working solutions before analysis.

After the sample was thawed and smashed, 0.05 g of the sample was mixed with 500 µL of 70% methanol. The sample was vortexed for 3 min under the condition of 2500 r/min and centrifuged at 12000 r/min for 10 min at 4 °C. Take 300 μL of supernatant into a new centrifuge tube and place the supernatant in -20 °C refrigerator for 30 min. The supernatant was centrifuged again at 12000 r/min for 10 min at 4°C. After centrifugation, transfer 200 μL of supernatant through a protein precipitation plate for further LC-MS analysis.

The sample extracts were analyzed using an LC-ESI-MS/MS system (Waters ACQUITY H-Class, https://www.waters.com/nextgen/us/en.html; MS, QTRAP® 6500+ System, <https://sciex.com/>). The analytical conditions were as follows. Amide method: HPLC: column, ACQUITY UPLC BEH Amide (i.d.2.1×100 mm, 1.7 μm); solvent system, water with 10 mM Ammonium acetate and 0.3% Ammonium hydroxide (A), 90% acetonitrile/water (V/V) (B); The gradient was started at 95% B (0-1.2 min), decreased to 70% B (8 min),50% B (9-11 min), finally ramped back to 95% B (11.1-15 min); flow rate: 0.4 mL/min; temperature, 40 °C; injection volume: 2 μL.

Linear ion trap and triple quadrupole scans were acquired on a triple quadrupole-linear ion trap mass spectrometer (QTRAP), QTRAP® 6500+ LC-MS/MS System, equipped with an ESI Turbo Ion-Spray interface, operating in both positive and negative ion mode and controlled by Analyst 1.6.3 software (Sciex). The ESI source operation parameters were as follows: ion source, ESI+/-; source temperature 550 °C; ion spray voltage (IS) 5500 V (Positive), -4500 V (Negative); curtain gas was set at 35 psi, respectively. Tryptophan and its metabolites were analyzed using scheduled multiple-reaction monitoring. Data acquisitions were performed using Analyst 1.6.3 software (Sciex). Multiquant 3.0.3 software (Sciex) was used to quantify all metabolites. Mass spectrometer parameters, including the DP and CE for individual MRM transitions, were done with further DP and CE optimization. A specific set of MRM transitions was monitored for each period according to the metabolites eluted within this period (8-10).

The hypergeometric test used in the enrichment analysis of KEGG pathways is calculated as follows:

$$P=1-\sum_{i=0}^{m-1} \frac{\left( \begin{matrix} M \\ i \end{matrix} \right)\left( \begin{matrix} N-M \\ n-i \end{matrix} \right)}{\left( \begin{matrix} N \\ n \end{matrix} \right)}$$

N represents the number of metabolites with KEGG annotations in all metabolites; n represents the number of differential metabolites in N; M represents the number of metabolites in a KEGG pathway in N; and m represents the number of differential metabolites in a KEGG pathway in M. The closer the *P*-value is to 0, the more significant the enrichment.

**References**

1. He Z, Du J, Zhang Y, Xu Y, Huang Q, Zhou Q, et al. Kruppel-like factor 2 contributes to blood-spinal cord barrier integrity and functional recovery from spinal cord injury by augmenting autophagic flux. Theranostics. 2023;13(2):849-66.

2. Chen W, Gong L, Guo Z, Wang W, Zhang H, Liu X, et al. A novel integrated method for large-scale detection, identification, and quantification of widely targeted metabolites: application in the study of rice metabolomics. Mol Plant. 2013;6(6):1769-80.

3. Fraga CG, Clowers BH, Moore RJ, Zink EM. Signature-discovery approach for sample matching of a nerve-agent precursor using liquid chromatography-mass spectrometry, XCMS, and chemometrics. Anal Chem. 2010;82(10):4165-73.

4. Medeiros PM, Simoneit BR. Analysis of sugars in environmental samples by gas chromatography-mass spectrometry. J Chromatogr. 2007;1141(2):271-8.

5. Zheng H, Zhang Q, Quan J, Zheng Q, Xi W. Determination of sugars, organic acids, aroma components, and carotenoids in grapefruit pulps. Food Chem. 2016;205:112-21.

6. Gomez-Gonzalez S, Ruiz-Jimenez J, Priego-Capote F, Luque de Castro MD. Qualitative and quantitative sugar profiling in olive fruits, leaves, and stems by gas chromatography-tandem mass spectrometry (GC-MS/MS) after ultrasound-assisted leaching. J Agric Food Chem. 2010;58(23):12292-9.

7. Sun S, Wang H, Xie J, Su Y. Simultaneous determination of rhamnose, xylitol, arabitol, fructose, glucose, inositol, sucrose, maltose in jujube (Zizyphus jujube Mill.) extract: comparison of HPLC-ELSD, LC-ESI-MS/MS and GC-MS. Chem Cent J. 2016;10:25.

8. Rathod R, Gajera B, Nazir K, Wallenius J, Velagapudi V. Simultaneous Measurement of Tricarboxylic Acid Cycle Intermediates in Different Biological Matrices Using Liquid Chromatography-Tandem Mass Spectrometry; Quantitation and Comparison of TCA Cycle Intermediates in Human Serum, Plasma, Kasumi-1 Cell and Murine Liver Tissue. NA. 2020;10(3).

9. Oeckl P, Ferger B. Simultaneous LC-MS/MS analysis of the biomarkers cAMP and cGMP in plasma, CSF and brain tissue. J Neurosci Methods. 2012;203(2):338-43.

10. Luo B, Groenke K, Takors R, Wandrey C, Oldiges M. Simultaneous determination of multiple intracellular metabolites in glycolysis, pentose phosphate pathway and tricarboxylic acid cycle by liquid chromatography-mass spectrometry. J Chromatogr. 2007;1147(2):153-64.
